# The *Rhipicephalus sanguineus* group: updated list of species, geographical distribution, and vector competence

**DOI:** 10.1186/s13071-024-06572-3

**Published:** 2024-12-27

**Authors:** Filipe Dantas-Torres, Lucas C. de Sousa-Paula, Domenico Otranto

**Affiliations:** 1 Department of Immunology, Aggeu Magalhães Institute, Oswaldo Cruz Foundation (Fiocruz), Recife, Brazil; 2 Tick-Pathogen Transmission Unit, Laboratory of Bacteriology, Division of Intramural Research, National Institute of Allergy and Infectious Diseases (NIAID), Hamilton, MT USA; 3 Department of Veterinary Medicine, University of Bari, Valenzano, Bari, Italy; 4 Department of Veterinary Clinical Sciences, City University of Hong Kong, Hong Kong, China

**Keywords:** *Rhipicephalus*, Ixodidae, Distribution, Vector, Pathogens

## Abstract

The *Rhipicephalus sanguineus* group is an assembly of species morphologically and phylogenetically related to *Rhipicephalus sanguineus* sensu stricto. The taxonomy and systematics of this species group have remained obscure for a long time, but extensive research conducted during the past two decades has closed many knowledge gaps. These research advancements culminated in the redescription of *R. sanguineus* sensu stricto, with subsequent revalidation of former synonyms (*Rhipicephalus linnaei*, *Rhipicephalus rutilus*, and *Rhipicephalus secundus*) and even the description of new species (*Rhipicephalus afranicus* and *Rhipicephalus hibericus*). With a much clearer picture of the taxonomy of these species, we present an updated list of species belonging to the *R. sanguineus* group, along with a review of their geographic distribution and vector role for various pathogens of animals and humans. We also identify knowledge gaps to be bridged in future studies.

**Graphical abstract:**

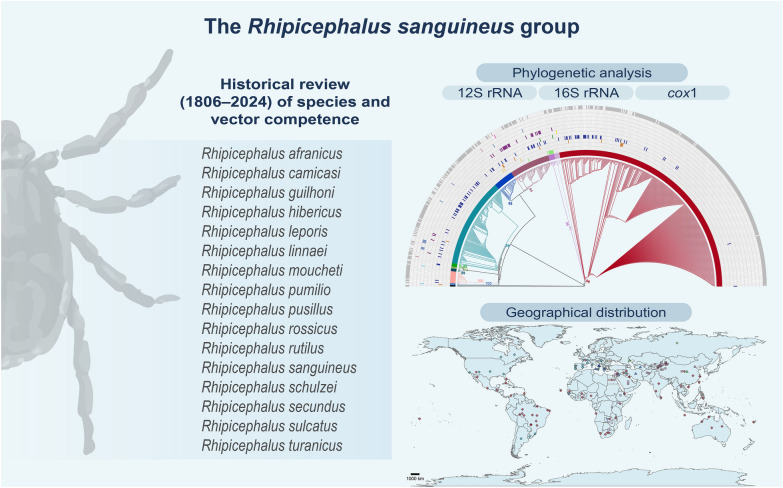

**Supplementary Information:**

The online version contains supplementary material available at 10.1186/s13071-024-06572-3.

## Background

The family Ixodidae Murray, 1877, presently includes ~ 786 tick species considered valid [1–14], 90 of which belong to the genus *Rhipicephalus* Koch, 1844 [15]. *Rhipicephalus* spp. ticks are commonly named “brown ticks” because of their characteristic brown colour, whose tonality and intensity vary from yellowish to dark brown. More rarely, they may be ornate [only four species, *Rhipicephalus pulchellus* (Gerstäcker, 1873); *Rhipicephalus dux* Dönitz, 1910; *Rhipicephalus humeralis* Tonelli Rondelli, 1926; and *Rhipicephalus maculatus* Neumann, 1901] and exhibit ivory ornamentation on their dorsal scutum in the adult stage [16].

*Rhipicephalus sanguineus* (Latreille, 1806) [17] [henceforth referred to as *R. sanguineus* sensu stricto (s.s.)] is the type species of the genus *Rhipicephalus*. This species was originally described as *Ixodes sanguineus* Latreille, 1806 [17], and was later ascribed to the genus *Rhipicephalus* by Koch [15]. In his ground-breaking work, Koch [15] described the genus *Rhipicephalus* and several new species, some of which (i.e. *Rhipicephalus siculus* Koch, 1844 [15], *Rhipicephalus limbatus* Koch, 1844 [15], *Rhipicephalus rutilus* Koch, 1844 [15]) were placed in synonym with *R. sanguineus* s.s. by Neumann [18].

Since its original description, *R. sanguineus* s.s., and related species, have been studied by several tick taxonomists based on specimens collected from different zoogeographical regions [15, 18–50]. To learn more about this history, see Nava et al. [51] and Dantas-Torres and Otranto [52].

In many of the abovementioned studies, the authors were unwittingly dealing with distinct taxa, some of which were mistakenly placed under the name “*R. sanguineus*”. This misconception was challenged by the authors of a series of crossbreeding experiments [53–56], refined morphological studies [55, 57–59] and more extensive phylogenetic analyses [53, 55, 58, 60–67] conducted during the past two decades, which culminated in the redescription of *R. sanguineus* s.s. [68]. Thus, what used to be called “*R. sanguineus*” in many parts of the world is not actually *R. sanguineus* s.s. but rather similar species that belong to the so-called *Rhipicephalus sanguineus* group. Considering the overlapping morphological features of some ticks belonging to this group, the use of the term “sensu lato” (s.l.) has been encouraged for ticks that morphologically resemble *R. sanguineus* s.s., but have not been assessed genetically, or have been found to be genetically distinct from the latter. In sum, these ticks should generally be referred to as *R. sanguineus* s.l. [68]. In the same way, ticks that morphologically resemble *Rhipicephalus turanicus* Pomerantzev, 1940, but have not been assessed genetically, or have been found to be genetically distinct from this species, are referred to as *R. turanicus* s.l. [68]. While these terms are informal from a taxonomic perspective, they are useful when expressing uncertainty about the actual identity of the ticks under study.

The *R. sanguineus* group was extensively studied in the pioneering works of the great tick taxonomists mentioned above, but several knowledge gaps persisted for decades, principally due to the absence of a name-bearing specimen for *R. sanguineus* s.s. and the unavailability of more recent tools, such as DNA sequencing. This enormous gap was bridged in 2018 with the designation of a neotype for *R. sanguineus* s.s., along with a complete morphological description of all of its developmental stages, and the generation of reference DNA sequences [68]. This fundamental work stimulated further taxonomic work, with the revalidation of old synonyms [69–72] and descriptions of new species [14, 73].

With a much clearer picture of the taxonomy of this important group of ticks, we have prepared an updated list of species belonging to the *R. sanguineus* group and discuss their geographical distribution and vector role for various pathogens. Finally, we identify knowledge gaps to be bridged in future studies.

## Definition of the *R. sanguineus* group

The *R. sanguineus* group was conceived as one that included *R. sanguineus* s.s. and morphologically related species. Morphological definitions of this species group have been proposed by Morel and Vassiliades [33] and Pegram et al. [42]. However, these morphological definitions are clearly insufficient for the separation of species belonging to the *R. sanguineus* group from those of other *Rhipicephalus* spp. Examples of generic morphological features that are not exclusive to species of this group include: males with “interstitial punctations variable in size and density”, “spiracular plates variable (but most useful diagnostic character)”, and “adanal plates usually twice as long as wide (but too variable intraspecifically to be of diagnostic value)” [42]. Some features of females include “scutum usually longer than wide” and “scutal punctation variable as in males”. These features do not pertain exclusively to the species included in the *R. sanguineus* group and therefore cannot be used to separate them from other congeners.

It is now well established that morphology is not always sufficient for the assessment of species’ relationships and boundaries, especially within a genus that includes numerous species with often overlapping morphological features. For example, *Rhipicephalus pusillus* Gil Collado, 1936 [74], which is part of the *R. sanguineus* group, was originally described as *Rhipicephalus bursa pusillus* [74] due to its morphological similarities with *Rhipicephalus bursa* Canestrini and Fanzago, 1878. Therefore, one could argue that *R. bursa* should also be included in the *R. sanguineus* group; however, it is not. Indeed, *R. pusillus* and *R. bursa* belong to different phylogenetic clades, with only the first being included in the *R. sanguineus* s.s. clade [75]. Similarly, *Rhipicephalus bergeoni* Morel and Balis, 1976 [38] was included in the *R. sanguineus* group by Morel and Balis [38] in their original species description, despite its morphological relationship with *Rhipicephalus appendiculatus* Neumann, 1901, which is not part of the *R. sanguineus* group.

Therefore, the *R. sanguineus* group should be defined as a group of species morphologically and, most importantly, phylogenetically related to *R. sanguineus* s.s. and included in its clade. In the following section, we list the species previously included in this species group and provide an updated list of species.

## Phylogenetic relationships within the *R. sanguineus* group

Species belonging to the *R. sanguineus* group form a well-supported clade which excludes other congeners, including *R. bergeoni* [75]. We conducted comprehensive analyses of 12S ribosomal RNA (rRNA), 16S rRNA and *cox1* gene sequences from all of the species belonging to the *R. sanguineus* group (except *Rhipicephalus schulzei* Olenev, 1929) available in GenBank (for methodological details, see Additional files 1, 2, 3, 4 and 5). These analyses congruently demonstrated the existence of well-defined clades within the *R. sanguineus* group. These clades include reference sequences (i.e. from original species descriptions, redescriptions, and taxonomic studies) and phylogenetically related sequences, which are not necessarily registered under the corresponding species name.

**Fig. 1:**
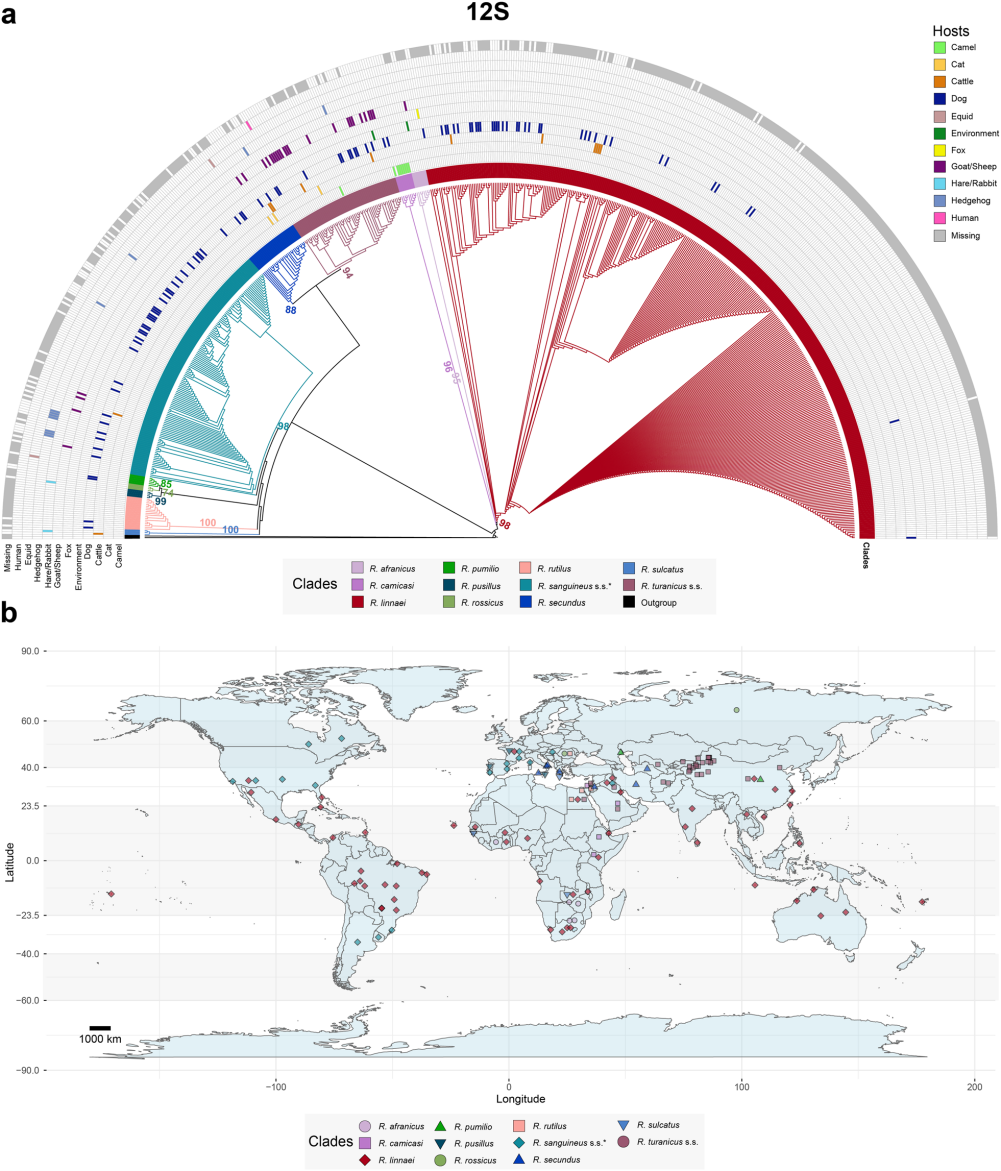
Global distribution and host association of the *Rhipicephalus sanguineus* group on the basis of 635 12S ribosomal RNA (rRNA) sequences available in GenBank. **a** Maximum-likelihood tree inferred via alignment with 391 sites and the TIM3 + F + G4 model. The coloured branches depict clades representing different members of the *R. sanguineus* group, with host associations indicated by coloured cells based on GenBank data. Bootstrap values > 50 supporting the origin nodes of each clade are shown. A comprehensive tree with all bootstrap values and sequence labels is available in Additional file 6. **b** Map illustrating the global distribution of the *R. sanguineus* group. Sequences clustered on the phylogenetic tree were used to generate the map. Coordinates from GenBank were deduplicated for each clade. Dots on the map represent approximate midpoints when precise location data (e.g. city-level data) were not available (e.g. “USA” instead of “USA: Los Angeles”). This map was created via R (version 4.3.0) with ggplot2 (version 3.4.4) and maps (version 3.4.1) packages

**Fig. 2:**
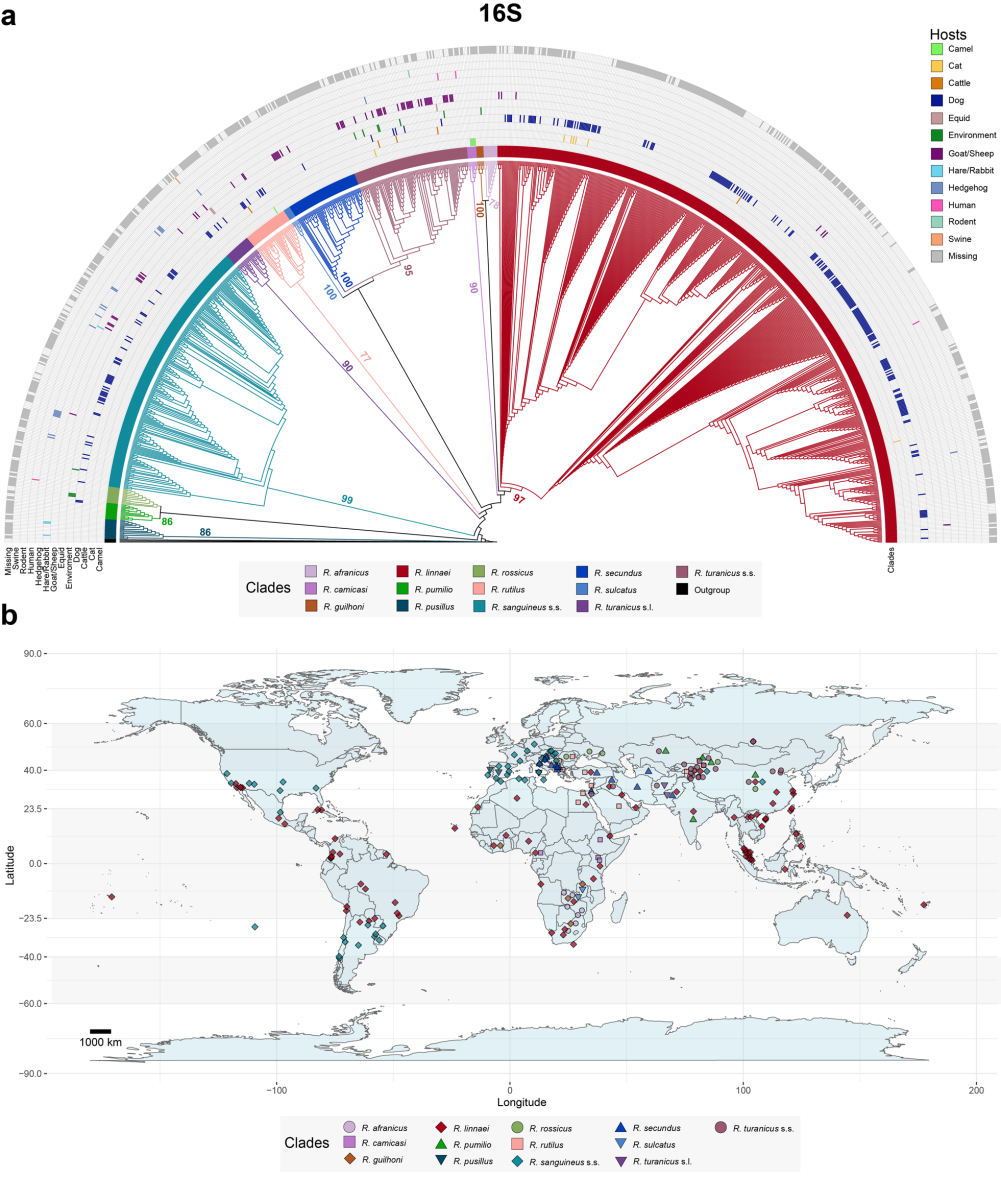
Global distribution and host association of the *Rhipicephalus sanguineus* group based on 1062 16S rRNA sequences available in GenBank. **a** Maximum-likelihood tree inferred via alignment with 526 sites and the TIM3 + F + R4 model. The coloured branches depict clades representing different members of the *R. sanguineus* group, with host associations indicated by coloured cells based on GenBank data. Bootstrap values > 50 supporting the origin nodes of each clade are shown. A comprehensive tree with all bootstrap values and sequence labels is available in Additional file 7. **b** Map illustrating the global distribution of the *R. sanguineus* group. Sequences clustered on the phylogenetic tree were used to generate the map. Coordinates from GenBank were deduplicated for each clade. Dots on the map represent approximate midpoints when precise location data (e.g. city-level data) were not available (e.g. “USA” instead of “USA: Los Angeles”). This map was created via R (version 4.3.0) with ggplot2 (version 3.4.4) and maps (version 3.4.1) packages

**Fig. 3:**
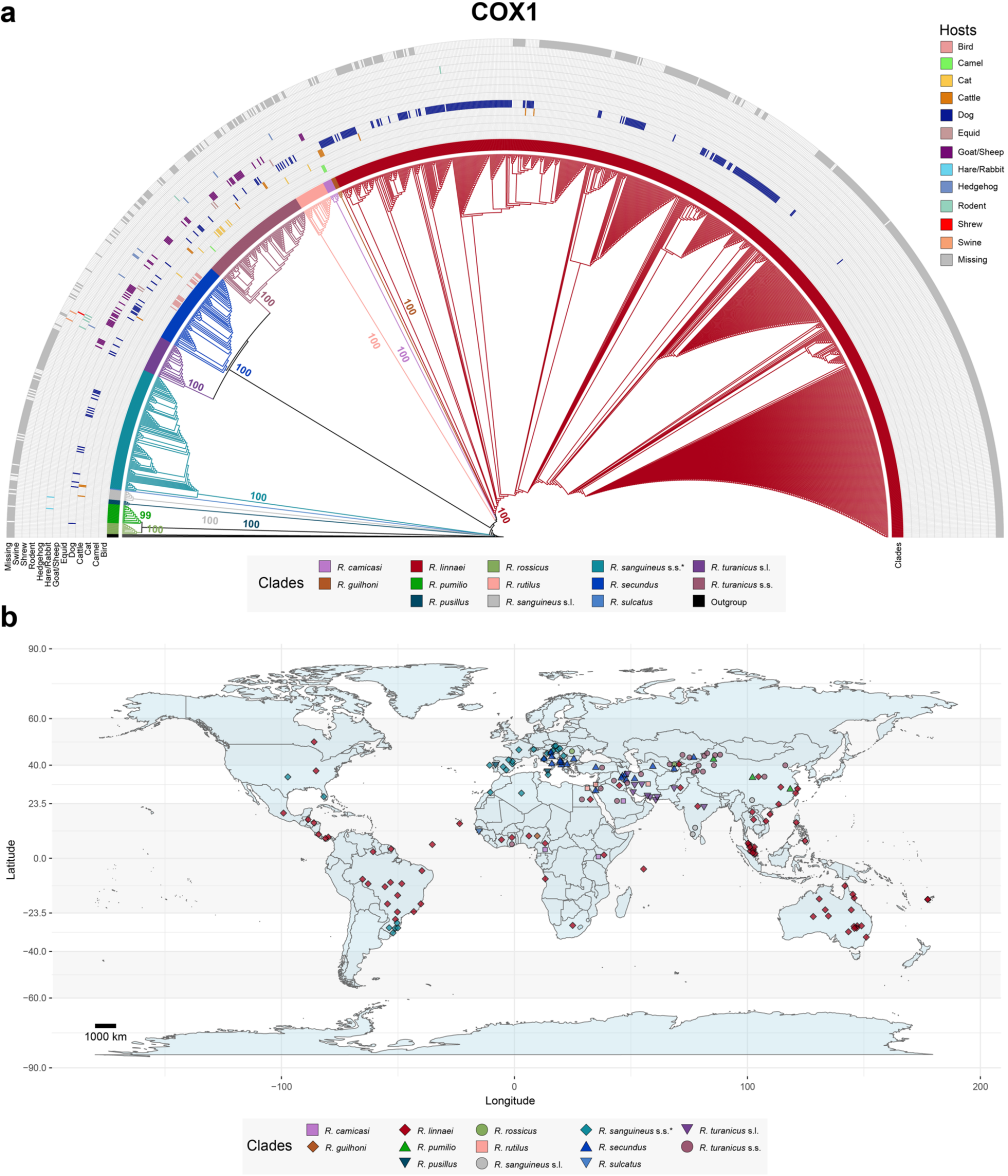
Global distribution and host association of the *Rhipicephalus sanguineus* group on the basis of 1115 *cox1* sequences available in GenBank. **a** Maximum-likelihood tree inferred via alignment with 644 sites and the TN + F + I + R3 model. The coloured branches depict clades representing different members of the *R. sanguineus* group, with host associations indicated by coloured cells based on GenBank data. Bootstrap values > 50 supporting the origin nodes of each clade are shown. A comprehensive tree with all bootstrap values and sequence labels is available in Additional file 8. **b** Map illustrating the global distribution of the *R. sanguineus* group. Sequences clustered on the phylogenetic tree were used to generate the map. Coordinates from GenBank were deduplicated for each clade. Dots on the map represent approximate midpoints when precise location data (e.g. city-level data) were not available (e.g. “USA” instead of "USA: Los Angeles”). This map was created via R (version 4.3.0) with ggplot2 (version 3.4.4) and maps (version 3.4.1) packages

Although an in-depth discussion is beyond the scope of this review, the phylogenetic analyses presented here provide a glimpse into the relationships between some species of the *R. sanguineus* group (Figs. 1, 2 and 3). For example, *Rhipicephalus rossicus* Yakimov and Kohl-Yakimova, 1911, and *Rhipicephalus pumilio* Schulze, 1935, have a common ancestor but form well-supported, distinct clades. As expected, not all of the included sequences of these clades were properly assigned to the corresponding species (see Additional files 6, 7 and 8). Taking the *cox1* tree (Fig. 3) as an example, a single sequence attributed to *R. pumilio* (accession no. AY008684) was positioned within the cluster representing the *R. rossicus* clade and may represent this species. These incongruences between the name registered in GenBank and the actual species are common and are expected to continue to arise, considering the complicated morphological identification of these closely related species, especially in countries where multiple members of this group coexist. This, however, should no longer be a major problem, considering the availability of reference sequences for most of these species [68–73, 75]. The inclusion of these reference sequences is therefore advocated for new studies generating new molecular data for the *R. sanguineus* group.

*Rhipicephalus turanicus* and *Rhipicephalus secundus* Feldman-Muhsam, 1952 [31] share a common ancestor with another clade, the “*R. turanicus* s.l. clade” (Figs. 2, 3), which includes ticks from India, Iran, Pakistan and Afghanistan. These ticks have been registered in GenBank as either “*R. sanguineus*” or “*R. turanicus*”, but clearly belong to a distinct lineage. Regarding *R. turanicus* and *R. secundus*, Dantas-Torres et al. [58] generated 12S rRNA, 16S rRNA and *cox1* sequences from ticks collected from the wild in Turkmenistan [Zoological Institute of the Russian Academy of Sciences (ZINRAS) no. 426682, date of collection 7 May 1967, locality Kara-Kala; collected by Y. S. Balashov]. These ticks were morphologically identified as *R. turanicus* by A. Filippova, who emphasized that this was a difficult, highly polymorphic species (personal communication with FDT and DO; St. Petersburg, 2013). Bakkes et al. [73] redescribed this species based on specimens from dogs in Turkmenistan, but Guglielmone et al. [9] argued that it would be premature to define this species without further morphological and molecular analyses of specimens collected from *Ovis aries* (type host) in Tashkent (type locality), Uzbekistan. The ticks from Turkmenistan studied by Dantas-Torres et al. [58] and Bakkes et al. [73] were, in fact, phylogenetically related to what is now defined as *R. secundus*, which also agrees with our current analyses (Additional files 6, 7 and 8). At the time these studies were conducted, *R. secundus* was still relegated to a synonym of *R. turanicus*. Analysing a larger number of sequences, Bakkes et al. [73] clearly found two well-supported clades, one with ticks from Turkmenistan, Italy and Greece and another one with ticks from Afghanistan, China, Kyrgystan and Israel, although they assigned both clades to the “*R. turanicus* Palearctic lineage”. Mumcuoglu et al. [72] reported similar results but considered the two clades as distinct taxa: *R. secundus* (Turkey, Corsica, Italy, Albania and Israel) and *R. turanicus* (Afghanistan, China, Kyrgystan, Israel and Uzbekistan). The phylogenetic analysis based on 12S rRNA gene sequences included a 339-base pair sequence (accession number FJ536579) of a tick collected from the type host (*O. aries*) in Uzbekistan, and therefore we tentatively follow Mumcuoglu et al. [72] here. While we consider *R. secundus* as a valid species (a position also adopted by Guglielmone et al. [9]), we emphasize that new, longer DNA sequences (e.g. the mitogenome) from *R. turanicus* collected from sheep in Uzbekistan are needed to generate reference sequences for this species. This will be a fundamental step in mitigating uncertainties regarding the actual geographical distribution of this species (see the discussion in Guglielmone et al. [9]).

A single 12S rRNA sequence (accession number FJ536557) attributed to *Rhipicephalus leporis* Pomerantzev, 1946 [United States National Tick Collection (USNTC)-Rocky Mountain Laboratory (RML) voucher 118356] was positioned in the cluster of the *Rhipicephalus linnaei* (Audouin, 1826) clade (Fig. 1). This sequence was nearly identical (99.7%; identities = 338/339) to the reference sequence of *R. linnaei* (accession number OM994391). Similarly, *cox1* sequences attributed to *R. leporis* also clustered together within the *R. linnaei* clade (Fig. 3), as reported by Hornok et al. [67]. As no bona fide reference sequence for *R. leporis* is currently available, further studies are needed to solve this puzzle. In fact, Guglielmone et al. [9] questioned reports of alleged *R. leporis* in Iraq, Iran, Kenya and the Ivory Coast, emphasizing that the uncertainties surrounding this species will be solved only by studying specimens collected from the type host at the type locality. The male lectotype of *R. leporis* was deposited in the ZINRAS Collection (St. Petersburg, Russia). Genetic data from this specimen, or from new specimens collected from hares in Kenimekh District, Uzbekistan, would be valuable for the generation of bona fide sequences (e.g. complete *cox1* or mitogenome) of *R. leporis* and to distinguish it from other members of the *R. sanguineus* group.

Available *cox1* sequences indicate the existence of a distinct group (“*R. sanguineus* s.l. clade”; Fig. 3) of ticks found in India and China, which are phylogenetically related, but separate from, a single sequence attributed to *Rhipicephalus sulcatus* Neumann, 1908, and from the *R. sanguineus* s.s. clade. The sequences included in the *R. sanguineus* s.l. clade were mostly labelled “*R. sanguineus*”, but also as “*R. rutilus*” or “*R. turanicus*” (Additional file 8). Further large-scale studies in these countries may be valuable for defining the identities of these ticks.

Our analyses revealed incongruent results regarding the phylogenetic position of *R. sulcatus* within the *R. sanguineus* group (Figs. 1, 2 and 3). These findings agree with those presented by Bakkes et al. [73, 75], who reported that the phylogenetic position of *R. sulcatus* within the *R. sanguineus* group varied in their consensus trees presented in 2020 and 2021. This was mostly probably a result of evolutionary inferences based on short, partial gene sequences, and should be resolvable by more robust analyses using, for instance, the complete mitogenome. Guglielmone et al. [76] considered the redescriptions of adults and descriptions of the immature *R. sulcatus* in Theiler and Robinson [77] provisional, and still treated this species as provisional in their last list [9], considering that its morphological separation from several other members of *R. sanguineus* s.s. is difficult. We agree that further molecular studies are needed to resolve the taxonomic problems associated with *R. sulcatus* and related ticks.

The recently described *Rhipicephalus hibericus* Millán, Rodríguez-Pastor and Estrada-Peña, 2024 [14] formed a monophyletic clade with *R. sanguineus* s.s. In their original description, Millán et al. [14] concluded that *R. hibericus* “is in a sister clade of *R. sanguineus* s.s.”, as inferred from *cox1* gene fragments. However, they only included a limited number of sequences from *R. sanguineus* s.s. and did not include reference sequences from Nava et al. [68]. In the legend of their Fig. 6, they wrote “Included are samples of *R. hibericus* n.sp., *R. sanguineus* s.s. (from the colony used for the redescription of the species) …”, but we could not identify any *R. sanguineus* s.s. sequences from France. Most *cox1* gene sequences of *R. hibericus* reported in Millán et al. [14] are nearly identical (99.5–99.7% identity) to the reference sequence of *R. sanguineus* s.s. from Montpellier (accession number MH630346). The phylogenetic trees inferred from 12S rRNA and 16S rRNA gene fragments included more sequences, including those from France. In these trees, *R. hibericus* sequences are included in the *R. sanguineus* s.s. clade. Nava et al. [68] also emphasized that ticks resembling *R. turanicus* from the western Mediterranean region of Europe clustered with *R. sanguineus* s.s., regardless of the mitochondrial gene used to infer the phylogeny (16S rRNA, 12S rRNA, *cox1*)*.* Our analyses, which included a larger number of *cox1*, 12S rRNA and 16S rRNA gene sequences (including those from Nava et al. [68]), also placed *R. hibericus* within the *R. sanguineus* s.s. clade (Figs. 1, 2 and 3). The very low base pair difference between *R. sanguineus* s.s. and *R. hibericus* and their monophyly are compatible with their placement in a single species (see further discussion in the “Knowledge gaps” section).

## Updated list of species belonging to the *R. sanguineus* group

Traditionally, 12 species have been included in the *R. sanguineus* group as follows (in order of description): *R. sanguineus* s.s.; *R. sulcatus*; *R. rossicus*; *R. schulzei*; *R. pumilio*; *R. pusillus*; *R. turanicus*; *R. leporis*; *Rhipicephalus guilhoni* Morel and Vassiliades, 1963 [33]; *Rhipicephalus moucheti* Morel, 1965 [78]; *R. bergeoni*; and *Rhipicephalus camicasi* Morel, Mouchet and Rodhain, 1976 [39, 42, 43]. Although Pegram et al. [43] questioned the inclusion of *R. bergeoni* in the *R. sanguineus* group, they still included this species in the group, as did Camicas et al. [79]. In our updated list (Table 1), we removed *R. bergeoni*, as it shows morphological and phylogenetic affinities with *R. appendiculatus* and is clearly separate from the *R. sanguineus* group clade [75].

**Table 1:** Updated list of species belonging to the *Rhipicephalus sanguineus* group (listed chronologically according to their first description)

Species and authorities	Source of reference DNA sequences
*Rhipicephalus sanguineus* (Latreille, 1806) [17]	Nava et al. [68] (redescription)
*Rhipicephalus linnaei* (Audouin, 1826)	Šlapeta et al. [70] (reinstatement)
*Rhipicephalus rutilus* Koch, 1844 [15]	Šlapeta et al. [71] (reinstatement)
*Rhipicephalus sulcatus* Neumann, 1908	
*Rhipicephalus rossicus* Yakimov and Kohl-Yakimova, 1911	
*Rhipicephalus schulzei* Olenev, 1929	
*Rhipicephalus pumilio* Schulze, 1935	
*Rhipicephalus pusillus* Gil Collado, 1936 [74]	Estrada-Peña et al. [95] (redescription)
*Rhipicephalus turanicus* Pomerantzev, 1940 [27]^a^	
*Rhipicephalus leporis* Pomerantzev, 1946 [29]	
*Rhipicephalus secundus* Feldman-Muhsam, 1952 [31]	Mumcuoglu et al. [72] (reinstatement)
*Rhipicephalus guilhoni* Morel and Vassiliades, 1963 [33]	
*Rhipicephalus moucheti* Morel, 1965 [78]	
*Rhipicephalus camicasi* Morel, Mouchet and Rodhain, 1976 [39]	
*Rhipicephalus afranicus* Bakkes, 2020 [73]	Bakkes et al. [73] (description)
*Rhipicephalus hibericus* Millán, Rodríguez-Pastor and Estrada-Peña, 2024 [14]^b^	Millán et al. [14] (description)

^a^There is a 339-base pair 12S ribosomal RNA (rRNA) gene sequence (accession number FJ536579) from a tick (collected from a type host in Uzbekistan), which has been considered by some authors [72] to be a possible bona fide sequence of *R. turanicus*

^b^Tentatively considered as valid herein, pending further morphological and phylogenetic studies. It may be conspecific with *R. sanguineus* sensu stricto (s.s.) (see the discussion in the section “Knowledge gaps”)

Camicas et al. [79] included additional species (i.e. *Rhipicephalus ziemanni* Neumann, 1904; *Rhipicephalus aurantiacus* Neumann, 1906; *Rhipicephalus boueti* Morel, 1957; *Rhipicephalus ramachandrai* Dhanda, 1966; *Rhipicephalus tetracornus* Kitaoka and Suzuki, 1983) in the *R. sanguineus* group, with no clear justification for this. The taxonomic status of some of these species (*R. aurantiacus* and *R. tetracornus*) has been questioned, but they are considered valid by Horak et al. [80], Guglielmone et al. [9, 81, 82], Dantas-Torres [83] and here. However, these species were not included in the *R. sanguineus* group by Pegram et al. [42, 43]. As of 12 August 2024, there were no DNA sequences available in GenBank for these species.

In recent years, five species morphologically and phylogenetically related to *R. sanguineus* s.s. (= temperate lineage, southern lineage) have been revalidated or newly described: *R. linnaei* (= tropical lineage, northern lineage), *R. rutilus* (= southeastern Europe lineage), *R. secundus* (formerly referred to as *R. turanicus* in Europe), *Rhipicephalus afranicus* Bakkes, 2020 [73] (formerly referred to as *R. turanicus* in Africa) and *R. hibericus* (ticks that are *R. turanicus*-like in the western Mediterranean region but are genetically indistinguishable from *R. sanguineus* s.s.) [14, 70–73]. These species are considered valid herein and are included in the *R. sanguineus* group. Nonetheless, further comprehensive morphological and phylogenetic studies on *R. hibericus* are recommended to properly differentiate it from *R. sanguineus* s.s. (see discussion in the “Knowledge gaps” section).

## Geographical distribution

**Fig. 4:**
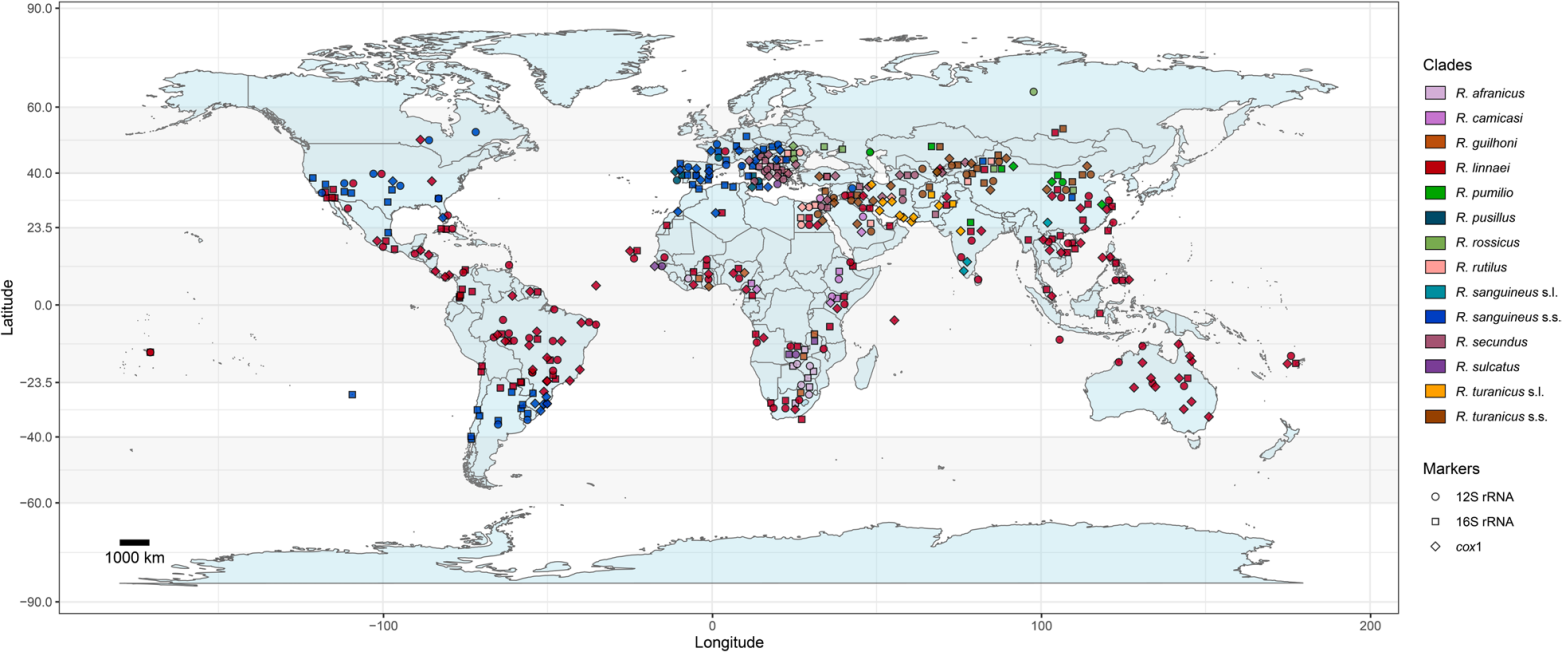
Map illustrating the global distribution of the *Rhipicephalus sanguineus* group based on all 12S rRNA, 16S rRNA and *cox1* sequences included in Figs. 1, 2 and 3. Colours depict the different clades of the *R*. *sanguineus* group and geometric forms represent the molecular markers. This map was created via R (version 4.3.0) with ggplot2 (version 3.4.4) and maps (version 3.4.1) packages

For many years, the distribution of *R. sanguineus* s.s. has been considered ubiquitous [84–86]. From a global perspective, the most widespread representatives of the *R. sanguineus* group are *R. sanguineus* s.s. and *R. linnaei*. The first predominates in temperate zones of the Nearctic, Neotropical and Palaearctic regions [9, 68], whereas the second is present mainly in tropical and subtropical areas of the Afrotropical, Australasian, Neotropical, Palaearctic, and Oriental regions (Figs. 1, 2, 3 and 4). Both species coexist in some areas, including Argentina, southern Brazil, Chile, northern Mexico, and the southern USA [87–90]. Locally, other members of the *R. sanguineus* group may predominate on dogs in Europe, Asia and Africa, as is the case for *R. secundus* in Basilicata, southern Italy [91]; *R. rossicus* in the Danube Delta, Romania [92, 93]; and *R. afranicus* in Huambo Province, Angola [94]. *Rhipicephalus rutilus* may also be more common than *R. sanguineus* s.s. in some areas of southeastern Europe [58, 66, 67]. Areas of sympatry between species may also occur in Europe, Asia and Africa, where different *Rhipicephalus* spp. (even outside the *R. sanguineus* group) may infest dogs. For example, in a study conducted in Tchicala-Tcholoanga, Huambo Province, Angola, the only members of the *R. sanguineus* group found on dogs were *R. afranicus* (referred to as *R. turanicus*; in 18 dogs) and *R. sulcatus* (in 14 dogs) [94]. However, several other *Rhipicephalus* spp. not belonging to the *R. sanguineus* group were found on dogs (number of infested dogs in parentheses): *Rhipicephalus decoloratus* Koch, 1844 [15] (*n* = 2); *Rhipicephalus lunulatus* Neumann, 1907 (*n* = 16); *Rhipicephalus punctatus* Warburton, 1912 (*n* = 9); *Rhipicephalus* *simus* Koch, 1844 [15] (*n* = 4); and *Rhipicephalus tricuspis* Dönitz, 1906 (*n* = 18). The morphological identification of *Rhipicephalus* spp. ticks may be a very difficult task in some regions of Eastern Europe, the Middle East, Asia, and Africa, where different species (even outside the *R. sanguineus* group) may occur.

The distributions of tick species belonging to the *R. sanguineus* group on the basis of nearly 2800 GenBank sequences analysed herein are shown in Figs. 1, 2, 3 and 4. The actual distribution of these species is certainly wider, as some sequences were excluded from the analyses (see Additional file 1) and because locality information was missing for many sequences deposited in GenBank (Additional files 9, 10 and 11). The sequences of *R. leporis* and *R. hibericus* are embedded in the *R. linnaei* and *R. sanguineus* s.s. clades, respectively (see Additional files 6, 7 and 8). The missing species include *R. moucheti* (sequences excluded from the analyses owing to their short length) and *R. schulzei* (no sequences available).

More detailed information on the distribution of all *R. sanguineus* group species is presented in Table 2. This information should also be interpreted with caution, considering the difficulties associated with the morphological determination of these species. Zoogeographical regions and countries are largely based on Guglielmone et al. [9], who performed a tremendous amount of work in compiling all of this information, and by also commenting on distribution records that require confirmation. Exceptions include the distributions of *R. hibericus*, *R. linnaei*, and *R. rutilus*, which were not compiled by Guglielmone et al. [9], and *R. turanicus*, for which we mostly follow Mumcuoglu et al. [72] (see a detailed discussion below). Unfortunately, Mumcuoglu et al. [72] did not include sequences from Turkmenistan, which may represent either *R. secundus* or a distinct species. Considering the uncertainties surrounding the identity of ticks from Turkmenistan, we did not include Turkmenistan in the distribution range of *R. secundus* or *R. turanicus*. Finally, doubtful records from Guglielmone et al. [9] are not listed herein.

**Table 2:** Geographical distribution of species belonging to the *Rhipicephalus sanguineus* group (listed alphabetically)

Species	Geographical distribution^a^
*Rhipicephalus afranicus*	Afrotropical—Angola, Botswana, Cameroon, Cote d’Ivoire (MK158983), Malawi, Mozambique, Namibia, Nigeria, Senegal, South Africa, Sudan, Tanzania, Uganda, Zambia, Zimbabwe [9, 73, 96, 97]
*Rhipicephalus camicasi*	Afrotropical—Cameroon (OK353734), Djibouti, Ethiopia, Kenya, Oman, Saudi Arabia (south), Somalia, Sudan, Yemen. Palearctic—Egypt, Jordan, Lebanon, Palestinian Territories, Saudi Arabia (north) [9, 16, 42, 44, 47, 96, 98–106]
*Rhipicephalus guilhoni*	Afrotropical—Burkina Faso, Cameroon, Central African Republic, Chad (south), Ethiopia, Mali (south), Mauritania (south), Niger (south), Nigeria, Senegal, South Sudan, Sudan. Palearctic—Chad (north), Egypt [9, 16, 42, 58, 78, 107–110]
*Rhipicephalus hibericus*	Palearctic—Portugal, Spain, southern France [14]^b^
*Rhipicephalus leporis*	Palearctic—Afghanistan, Kazakhstan, Tajikistan, Turkmenistan, Uzbekistan [9, 46, 86, 111]
*Rhipicephalus linnaei*	Afrotropical—Angola, Burkina Faso, Cameron, Cape Verde, Cote d'Ivoire, Djibouti, Ghana, Kenya, Malawi, Nigeria, Senegal, Seychelles, South Africa, Tanzania, United Arab Emirates, Zambia. Australasian—Australia, Fiji, Philippines. Neotropical—Argentina (north), Aruba, Belize, Brazil, Chile (north), Colombia, Costa Rica, Cuba, French Guiana, Grenada, Guatemala, Honduras, Mexico (south), Panama, Paraguay. Nearctic—Canada (likely imported, unlikely to be established), Mexico, USA, Venezuela. Palaearctic—Algeria, China, Croatia (likely imported, unlikely to be established), Egypt, France (likely imported, unlikely to be established), Iraq, Kuwait, Morocco, Russia (likely imported, unlikely to be established), Turkey. Oceania (Pacific)—American Samoa. Oriental—China, India, Indonesia (west of Wallace’s Line), Laos, Malaysia, Myanmar, Pakistan, Singapore, Sri Lanka, Taiwan, Thailand, Uzbekistan, Vietnam [53, 55, 60–67, 69, 70, 89, 112, 112–119]
*Rhipicephalus moucheti*	Afrotropical—Guinea, Benin, Cameroon, Sierra Leone [9, 16, 86, 120, 121]
*Rhipicephalus pumilio*	Oriental—China (south), India (MK621323, MK621324). Palearctic—China (north), Kazakhstan, Kyrgyzstan, Mongolia, Russia, Tajikistan, Turkmenistan, Uzbekistan [9, 16, 46, 86, 111, 122–126]
*Rhipicephalus pusillus*	Palearctic—France, Italy, Morocco, Portugal, Spain, Tunisia [9, 16, 33, 86, 103, 127, 128]
*Rhipicephalus rossicus*	Palearctic—Armenia, Azerbaijan, Bulgaria, China (north), Egypt, Georgia, Iran, Israel, Kazakhstan, Moldova, Romania, Russia, Turkey, Turkmenistan, Ukraine, Uzbekistan [9, 16, 46, 86, 103, 111, 129, 129–140]
*Rhipicephalus rutilus*	Nearctic—Canada (imported from Egypt, unlikely to be established). Palearctic—Egypt (Abbassia), Greece, Cyprus, Iran, Israel, Italy, Palestinian Territories, Romania, Serbia, Turkey [58, 67, 71, 113, 141]
*Rhipicephalus sanguineus* s.s.	Nearctic—Canada (imported, unlikely to be established), Mexico (north), USA. Neotropical—Argentina, Brazil (south), Chile, Uruguay. Oriental—Taiwan. Palearctic—Algeria, Canary Islands, China (north), Croatia, France, Germany, Hungary, Italy, Iraq, Malta, Morocco, Portugal, Serbia, Slovakia, Slovenia, Spain, Switzerland, Tunisia [58, 63, 68, 89, 141–145]
*Rhipicephalus schulzei*	Palearctic—Azerbaijan, China (north), Iran, Kazakhstan, Kyrgyzstan, Russia, Turkmenistan, Uzbekistan [9, 16, 46, 86, 107, 111, 124, 126, 132, 138, 140]
*Rhipicephalus secundus*	Palearctic—Albania, France, Greece, Israel, Italy, Palestinian Territories, Turkey [9, 58, 72]
*Rhipicephalus sulcatus*	Afrotropical—Angola, Benin, Botswana, Burkina Faso, Burundi, Cameroon, Central African Republic, Chad (south), Congo, Democratic Republic of the Congo, Eritrea, Ethiopia, Gabon, Ghana, Guinea, Guinea-Bissau, Ivory Coast, Kenya, Malawi, Mali (south), Mozambique, Namibia, Nigeria, Rwanda, Senegal, South Africa, South Sudan, Sudan, Tanzania, Togo, Uganda, Zambia, Zimbabwe [9, 16, 33, 42, 107, 110, 146–149]
*Rhipicephalus turanicus*^c^	Palearctic—Afghanistan, China (north), Israel, Kyrgyzstan, Uzbekistan [9, 72, 150]

^a^Regions and countries were mostly compiled from Guglielmone et al. [9] and references therein. The distribution of some species (e.g. *R. sanguineus* and *R. linnaei*) is much greater than reported, but we only include countries for which there are convincing genetic data

^b^These distribution data are in accordance with Millán et al. [14] but require confirmation. See the text for more details on the validity of this species and its presence in Portugal and France

^c^This species probably has a wider distribution in the Palearctic, but further genetic data are needed to confirm its presence in various countries where it was reported in the past

## Vector competence

Ticks belonging to the *R. sanguineus* group are important vectors of pathogens of clinical significance to domestic animals and humans [151]. More rarely, they have been implicated in cases of tick paralysis in dogs, both experimentally [152] and under field conditions [153].

In Table 3, we compiled information from numerous studies assessing the role of ticks belonging to the *R. sanguineus* group as vectors of various pathogens. While *R. sanguineus* s.s. is recognized as a significant vector of pathogens to dogs [154], many studies assessing the role of “*R. sanguineus*” as vectors were, in fact, dealing with different taxa. While studies conducted with ticks collected in tropical regions have focused mostly on *R. linnaei*, in subtropical and temperate regions, the situation is more complex, considering the variety of species that may be found on dogs (e.g. *R. rutilus*, *R. sanguineus* s.s., and *R. secundus*) in these regions. We tried our best to identify, with a certain level of certainty, the actual tick species to which these studies referred. However, for many old studies, it is virtually impossible to ascertain the actual species the authors were handling, either owing to uncertainties about the geographical origin of the ticks used in the experiments or because different species may be present in regions from which the ticks came. For example, this is the case for studies conducted with ticks from Texas [155, 156] and Arizona [157], where *R. linnaei* and *R. sanguineus* s.s. coexist [90, 158]. This also applies to studies conducted in Israel [159–162], where different species may parasitise dogs [71, 72].

**Table 3:** Studies assessing the role of *Rhipicephalus sanguineus* group ticks as vectors of pathogens

Study reference^a^	Year	Pathogen^b^	Reported tick species	Actual/putative tick species^c^	Tick origin	Insights on transmission results^d^
Christophers [163, 164]	1907	*Babesia vogeli*	*Rhipicephalus sanguineus*	*Rhipicephalus linnaei*?	Chennai, India	Transmission to dogs by bite; transstadial and transovarial transmission
Christophers [165]	1907	*Hepatozoon canis*	*R. sanguineus*	*R. linnaei*?	Chennai, India	Acquisition from naturally infected dogs
Christophers [166]	1912	*Hepatozoon canis*	*R. sanguineus*	*R. linnaei*?	Chennai, India	Acquisition from naturally infected dogs; transstadial transmission
Christophers [166]	1912	*Babesia vogeli*	*R. sanguineus*	*R. linnaei*?	Chennai, India	Transmission to dogs by bite; transstadial and transovarial transmission
Brumpt [167]	1919	*Babesia vogeli*	*R. sanguineus*	*R. sanguineus* s.s.?	Tunisia	Transmission to dogs by bite; transstadial and transovarial transmission
Rao [168], cited in Sen [169]	1926	*Babesia gibsoni*	*R. sanguineus*	*R. linnaei*?	Chennai, India	Unsuccessful transmission
Wenyon [170]	1926	*Babesia vogeli*	*R. sanguineus*	Several possible species	Aleppo, Syria	Transmission to dogs (by bite?)
Blanc and Caminopetros [171]	1930	*Leishmania infantum*	*R. sanguineus*	*R. sanguineus* s.s.?	France?	Acquisition from a naturally infected dog and from experimentally infected sousliks (*Spermophilus citellus*). Transmission to sousliks via inoculation of tick macerates; transstadial transmission
Durand and Conseil [172]	1930	*Rickettsia conorii*	*R. sanguineus*	*R. sanguineus* s.s.?	Tunisia or Marseille, France	Transmission to humans via inoculation of tick macerates
Mooser and Dummer [173]	1930	*Rickettsia typhi*	*R. sanguineus*	*R. sanguineus* s.s. or *R. linnaei*?	USA	Unsuccessful transmission
Rees [174]	1930	*Anaplasma marginale*	*R. sanguineus*	*R. sanguineus* s.s. or *R. linnaei*?	New Orleans, USA	Transmission to cattle by bite; transstadial transmission
Durand [175]	1931	*Rickettsia conorii*	*R. sanguineus*	*R. sanguineus* s.s.?	Tunisia	Transmission to humans via inoculation of tick macerates
Joyeux and Pieri [176]	1931	*Rickettsia conorii*	*R. sanguineus*	*R. sanguineus* s.s.?	Marseille, France	Transmission to humans via inoculation of tick macerates
Nieschulz and Wawo-Roentoe [177], cited in Brumpt [178]	1931	*Babesia canis*	*R. sanguineus*	*R. linnaei*?	Java, Indonesia	Unsuccessful transmission
Wenyon [179]	1931	*Hepatozoon canis*	*R. sanguineus*	*R. sanguineus* s.s. or *R. linnaei*?	Baghdad, Iraq	Transmission to dogs either fed or inoculated subcutaneously with emulsified tick organs
Blanc and Caminopetros [180]	1932	*Rickettsia conorii*	*R. sanguineus*	Several possible species	Greece	Transmission to humans via inoculation of tick macerates from some locations (e.g. Volo and Pireu), but not from others (e.g. Athens); transstadial and transovarial transmission
Brumpt [181]	1932	*Rickettsia conorii*	*R. sanguineus*	*R. sanguineus*?	Marseille, France	Transmission to humans and guinea pigs via inoculation of tick macerates; transstadial and transovarial transmission
Regendanz and Reichenow [182]	1932	*Mycoplasma haemocanis*	*R. sanguineus*	*R. sanguineus*?	Germany?	Unsuccessful transmission
Regendanz and Reichenow [183]	1932	*Babesia canis*	*R. sanguineus*	*R. linnaei*?	Java, Indonesia	Unsuccessful transmission
Parker et al. [155]	1933	*Rickettsia rickettsii*	*R. sanguineus*	*R. sanguineus* or *R. linnaei*?	Texas, USA	Transmission to guinea pig by bite; transstadial and transovarial transmission
Sen [169]	1933	*Babesia gibsoni*	*R. sanguineus*	*R. linnaei*?	India	Transstadial transmission
Rees [184]	1934	*Anaplasma marginale*	*R. sanguineus*	*R. sanguineus* or *R. linnaei*?	USA	Transmission to cattle by bite; transstadial transmission
Reichenow [185]	1935	*Babesia vogeli*	*R. sanguineus*	*R. sanguineus*?	Germany	Transmission to dogs by bite; transstadial and transovarial transmission
Roberts [186]	1935	*Rickettsia conorii*	*R. sanguineus*	Several possible species	Kenya	Transmission to guinea pigs via intraperitoneal inoculation of emulsified ticks (females plus eggs and females plus nymphs)
Regendanz and Muniz [187]	1936	*Babesia vogeli*	*R. sanguineus*	*R. linnaei*	Rio de Janeiro, Brazil	Transmission to dogs by bite; transstadial and transovarial transmission
Shortt [188]	1936	*Babesia vogeli*	*R. sanguineus*	*R. linnaei*?	India	Transmission to dogs by bite; transstadial and transovarial transmission
Agrinski [189]	1937	*Babesia equi*	*R. sanguineus*	Several possible species	Central Asia	Transmission to horses by bite
Donatien and Lestoquard [190]	1937	*Ehrlichia canis*	*R. sanguineus*	*R. sanguineus*?	Algeria	Transmission to dogs and macaques (*Macaca sylvanus*) via inoculation of tick macerates; transovarial transmission^e^
Fotheringham and Lewis [191]	1937	*Theileria parva*	*R. sanguineus*	Several possible species	Kenya?	Unsuccessful transmission
Brumpt [178]	1938	*Babesia rossi*	*R. sanguineus*	*R. sanguineus*?	Tunisia?	Unsuccessful transmission
Neitz and Thomas [192]	1938	*Ehrlichia canis*	*R. sanguineus*	*R. linnaei*?	Onderstepoort, South Africa	Transmission to black-backed jackals by bite
Neitz and Thomas [192]	1938	*Babesia vogeli*	*R. sanguineus*	*R. linnaei*?	Onderstepoort, South Africa	Transmission to black-backed jackals by bite
Rees and Avery [193]	1939	*Anaplasma marginale*	*R. sanguineus*	*R. sanguineus* or *R. linnaei*?	USA	Transovarial transmission
Datta [194]	1940	*Babesia gibsoni*	*R. sanguineus*	*R. linnaei*?	India	Transmission to dogs by bite; transstadial transmission
Kurchatov and Markov [195], cited in Neitz [196]	1940	*Babesia trautmanni*	*R. turanicus*	*R. turanicus*?	Russia	Transmission to pigs by bite; transstadial and transovarial transmission
Neitz et al. [197]	1941	*Rickettsia conorii*	*R. sanguineus*	Several possible species	South Africa	Transmission to guinea pigs by bite; transstadial and transovarial transmission
Smith [198]	1941	*Coxiella burnetii*	*R. sanguineus*	*R. linnaei*	Australia	Transmission to guinea pigs by bite and by inoculation of tick faeces; transstadial transmission
Enigk [199], cited in Neitz [196]	1944	*Babesia caballi*	*R. sanguineus*	Several possible species	Greece	Transmission to horses by bite; transstadial and transovarial transmission
Enigk [200]	1943	*Babesia caballi*	*R. sanguineus*	Several possible species	Tripoli, North Africa	Transmission to horses by bite; transstadial and transovarial transmission
Enigk [200]	1943	*Theileria equi*	*R. sanguineus*	Several possible species	Tripoli, North Africa	Transmission to horses by bite; transstadial transmission
Blanc et al. [201]	1946	*Coxiella burnetii*	*R. sanguineus*	*R. sanguineus* or *R. linnaei*?	Morocco	Acquisition from guinea pig and hedgehog by bite; transstadial transmission
Steinhaus [202], cited in Neiz [196]	1947	*Babesia vogeli*	*R. sanguineus*	*R. sanguineus* or *R. linnaei*?	USA	Transmission to dogs by bite; transstadial and transovarial transmission
Parker and Sussman [156]	1949	*Coxiella burnetii*	*R. sanguineus*	*R. sanguineus* or *R. linnaei*?	Arizona, USA	Transmission to guinea pigs via subcutaneous and intraperitoneal injection of tick macerates
Perez Gallardo et al. [203]	1949	*Coxiella burnetii*	*R. sanguineus*	*Rhipicephalus sanguineus* or *Rhipicephalus hibericus*?	Madrid, Spain	Transmission to guinea pigs via inoculation of tick macerates
Callot et al. [204]	1950	*Coxiella burnetii*	*R. sanguineus*	*R. sanguineus*?	Strasbourg, France	Transmission to guinea pigs by bite and inoculation of either tick macerates or tick faeces; transstadial transmission
Mantovani and Benazzi [205]	1953	*Coxiella burnetii*	*R. sanguineus*	Several possible species	Teramo, Italy	Transmission to guinea pigs via inoculation of extracts of engorged ticks and tick eggs (transovarial transmission?)
Shatas and Bystrova [206]	1954	*Francisella tularensis*	*Rhipicephalus rossicus*	*R. rossicus*?	Russia	Transmission to mice via by bite; transstadial transmission
Nel'zina et al. [207]	1960	*Yersinia pestis*	*Rhipicephalus schulzei*	*R. schulzei*?	Russia	Acquisition from infected laboratory animals; *Y. pestis* found in female tick intestine and its excrements; no transovarial or transstadial transmission
Petrov [208]	1960	*Francisella tularensis*	*R. rossicus*	*R. rossicus*?	Russia	Transmission to voles, lemmings, and guinea pigs by bite and tick ingestion; transstadial transmission
Bain [209]	1972	*Acanthocheilonema dracunculoides*	*R. sanguineus*	*R. sanguineus*?	Algeria	Third-stage larvae in naturally infected ticks
Senevtratna et al. [210]	1973	*Mycoplasma haemocanis*	*R. sanguineus*	*R. linnaei*?	Sri Lanka	Transmission to dogs by bite; transstadial and transovarial transmission
Kotel’nikova and Kondrashova [211]	1974	West Nile virus	*R. rossicus*	*R. rossicus*?	Russia	Acquisition from infected laboratory animals; transovarial and transstadial transmission
Burgdorfer et al. [212]	1975	*Rickettsia rhipicephali* ^f^	*R. sanguineus*	*R. sanguineus* or *R. linnaei*?	Mississippi, USA	Transmission to meadow voles (*Microtus pennsylvanicus*) and guinea pigs via intraperitoneal injection
Groves et al. [213]	1975	*Ehrlichia canis*	*R. sanguineus*	*R. linnaei*?	Vietnam	Transmission to dogs by bite; transstadial transmission
Groves et al. [213]	1975	*Ehrlichia canis*	*R. sanguineus*	*R. sanguineus* or *R. linnaei*?	Rocky Mountain Laboratory colony (USA origin?)	Transmission to dogs by bite; transstadial transmission
Kondratenko [214]	1976	Crimean haemorrhagic fever virus	*R. rossicus*	*R. rossicus*?	Russia	Transmission to little ground squirrels (*Spermophilus pygmaeus*) and rabbits by bite; transstadial and transovarial transmission
Smith et al. [215]	1976	*Ehrlichia canis*	*R. sanguineus*	*R. sanguineus* or *R. linnaei*?	Rocky Mountain Laboratory colony (USA origin?)	Transmission to dogs via by bite and inoculation; transstadial transmission
Lewis et al. [216]	1977	*Ehrlichia canis*	*R. sanguineus*	*R. sanguineus* or *R. linnaei*?	Laboratory colony (USA origin?)	Transmission to dogs by bite and inoculation; transstadial transmission
Kotel'nikova [217]	1978	West Nile virus	*R. rossicus*	*R. rossicus*?	Russia	Transstadial and transovarial transmission
Vanag and Grokhovskaia [218]	1978	Rabies virus	*R. sanguineus*	Several possible species	Russia	Transmission to mice by bite; transovarial and transstadial transmission
Liebisch and Gillani [219]	1979	*Babesia vogeli*	*R. sanguineus*	Several possible species	Cairo, Egypt	Transmission to dogs by bite
Liebisch and Gillani [219]	1979	*Babesia vogeli*	*R. sanguineus*	*R. sanguineus*?	Hannover, Germany	Transmission to dogs by bite; transstadial and transovarial transmission
Parker and Wilson [220]	1979	*Anaplasma marginale*	*R. sanguineus*	*R. linnaei*	Australia	Transmission to cattle by bite; transstadial transmission
Hafez et al. [221]	1981	*Theileria ovis*	*Rhipicephalus turanicus*	Several possible species	Egypt	No transstadial to sheep by bite; no transstadial transmission
Bain et al. [222]	1982	*Cercopithifilaria grassii*	*R. sanguineus*	*R. sanguineus*?	Switzerland (presumably originally transported from the south of France or Toscana)	Third-stage larvae in naturally infected ticks
Hafez et al. [223]	1982	*Babesia ovis*	*R. turanicus*	Several possible species	Egypt	Acquisition from infected sheep and evidence of transovarial transmission; no transmission to sheep by bite; no transstadial transmission
Parker [224]	1982	*Anaplasma marginale*	*R. sanguineus*	*R. linnaei*	Australia	Transmission to cattle by bite; transstadial transmission
Moltmann et al. [225]	1983	*Theileria equi*	*R. turanicus*	Several possible species	Northern Egypt	Acquisition from experimentally infected horses; transstadial transmission
Nordgren and Craig [226]	1984	*Hepatozoon canis*	*R. sanguineus*	*R. sanguineus* or *R. linnaei*?	Laboratory colony (USA origin?)	Transmission to dogs via oral ingestion of infected ticks; transstadial transmission
Bain et al. [227]	1986	*Cercopithifilaria roussilhoni*	*R. sanguineus*	*R. sanguineus* s.s.?	Montpellier, France	Third-stage larvae in nymphs, experimentally infected as larvae; transstadial transmission
Bain et al. [227]	1986	*Cercopithifilaria roussilhoni*	*R. sanguineus*	Several possible species	Niamey, Niger	Third-stage larvae in nymphs, experimentally infected as larvae; transstadial transmission
Bain et al. [227]	1986	*Cercopithifilaria roussilhoni*	*R. sanguineus*	Several possible species	Missira, Mali	Third-stage larvae in nymphs, experimentally infected as larvae; transstadial transmission
Petit et al. [228]	1988	*Monanema martini*	*R. sanguineus*	*R. sanguineus* s.s.?	Montpellier, France	Third-stage larvae in nymphs, experimentally infected as larvae; transstadial transmission
Petit et al. [228]	1988	*Monanema martini*	*R. turanicus*	*R. turanicus* or *R. secundus*?	Israel	Third-stage larvae in nymphs, experimentally infected as larvae; transstadial transmission
Petit et al. [229]	1988	*Cercopithifilaria roussilhoni*	*R. sanguineus*	*R. sanguineus* s.s.?	Montpellier, France	Third-stage larvae in nymphs, experimentally infected as larvae; transstadial transmission
Petit et al. [229]	1988	*Cercopithifilaria roussilhoni*	*R. sanguineus*	Several possible species	Missira, Mali	Third-stage larvae in nymphs, experimentally infected as larvae; transstadial transmission
Simpson et al. [230]	1991	*Anaplasma platys*	*R. sanguineus*	*R. sanguineus* s.s. or *R. linnaei*?	Obtained from Oklahoma State University (USA origin?)	Unsuccessful transmission
Potgieter et al. [231]	1992	*Babesia caballi*	*R. turanicus*	*Rhipicephalus afranicus*?	South Africa	No transstadial transmission
Potgieter et al. [231]	1992	*Theileria equi*	*R. turanicus*	*R. afranicus*?	South Africa	No transstadial transmission
Olmeda-García et al. [232]	1993	*Acanthocheilonema dracunculoides*	*R. sanguineus*	Several possible species	Laboratory colony (Spanish origin)	Transmission to dogs by bite; transstadial transmission
Yamane et al. [233]	1993	*Babesia gibsoni*	*R. sanguineus*	*R. sanguineus* s.s. or *R. linnaei*?	Laboratory colony (USA origin?)	Sporozoites in salivary glands of nymphs fed as larvae; unsuccessful transmission
Lewis et al. [234]	1996	*Babesia rossi*	*R. sanguineus*	*R. linnaei*?	Pretoria, South Africa	Unsuccessful transmission
Mathew et al. [235]	1996	*Ehrlichia canis*	*R. sanguineus*	*R. sanguineus* s.s. or *R. linnaei*?	Obtained from the Oklahoma State University (USA origin?)	Transmission to dogs by bite; transstadial transmission
Vincent-Johnson et al. [236]	1997	*Hepatozoon americanum*	*R. sanguineus*	*R. sanguineus* s.s. or *R. linnaei*?	Obtained from EL Laboratories Soquel, California (USA origin?)	No evidence of infection in ticks fed on infected dogs
Baneth et al. [159]	2001	*Hepatozoon canis*	*R. sanguineus*	Several possible species	Laboratory colony (Israeli origin?)	Transmission to dogs via oral ingestion of infected ticks; transstadial transmission
Ewing et al. [237]	2002	*Hepatozoon americanum*	*R. sanguineus*	*R. sanguineus* s.s. or *R. linnaei*?	Supplied by Oklahoma Agricultural Experiment Station (USA origin?)	No oocysts found in ticks fed on naturally or experimentally infected dogs
Razmi et al. [238]	2002	*Babesia ovis*	*R. sanguineus*	Several possible species	Iran	Kinetes morphologically resembling *B. ovis* in the haemolymph and two eggs of *R. sanguineus* sense lato (s.l.); sheep in the study areas were also infected by *B. motasi*
Stich et al. [239]	2002	*Ehrlichia canis*	*R. sanguineus*	*R. sanguineus* s.s. or *R. linnaei*?	Purchased from Oklahoma State University (USA origin?)	Transmission to guinea pigs by bite; transstadial transmission
Coutinho et al. [240]	2005	*Leishmania infantum*	*R. sanguineus*	*R. linnaei*	Minas Gerais, Brazil	Transmission to hamsters inoculated orally and peritoneally
Bremer et al. [241]	2005	*Ehrlichia canis*	*R. sanguineus*	*R. sanguineus* s.s. or *R. linnaei*?	Purchased from Oklahoma State University (USA origin?)	Transmission to dogs by bites of male ticks, which acquired the infection as nymphs (transstadial transmission), or as males
Forlano et al. [242]	2005	*Hepatozoon canis*	*R. sanguineus*	*R. linnaei*	Rio de Janeiro, Brazil	No transmission to a dog via oral administration of macerates from field-collected ticks; no evidence of infection in these ticks
Matsumoto et al. [243]	2005	*Rickettsia massiliae*	*R. turanicus*	*R. sanguineus* s.s.	Corsica, France	Transstadial and transovarial transmission
Matsumoto et al. [244]	2005	*Rickettsia conorii*	*R. sanguineus*	*R. linnaei*	Thailand	Transstadial and transovarial transmission
Baneth et al. [160]	2007	*Hepatozoon canis*	*R. sanguineus*	Several possible species	Laboratory colony (Israeli origin?)	Transmission to dogs via oral ingestion of infected ticks; transstadial transmission
Labruna et al. [245]	2008	*Rickettsia rickettsii*	*R. sanguineus*	*R. linnaei*	São Paulo, Brazil	Acquisition from guinea pigs; transstadial transmission
Levin et al. [161]	2009	*Rickettsia conorii*	*R. sanguineus*	*R. sanguineus* s.s. or *R. linnaei*?	Oklahoma, USA^g^	Transstadial transmission of *Rickettsia conorii israelensis*, but not of *Rickettsia conorii conorii*
Levin et al. [161]	2009	*Rickettsia conorii*	*R. sanguineus*	Several possible species	Israel	Transstadial transmission of *R. conorii israelensis*, but not of *R. conorii conorii*
Reichard et al. [246]	2009	*Cytauxzoon felis*	*R. sanguineus*	*R. sanguineus* s.s. or *R. linnaei*?	Purchased from Oklahoma State University Tick Rearing Facility (Stillwater, OK)	Unsuccessful transmission
Shkap et al. [162]	2009	*Anaplasma centrale*	*R. sanguineus*	Several possible species	Laboratory colony (Israeli origin?)	Acquisition from infected calves, but unsuccessful transmission to naïve ones
Shkap et al. [162]	2009	*Anaplasma marginale*	*R. sanguineus*	Several possible species	Laboratory colony (Israeli origin?)	Transmission to calves by bite
Socolovschi et al. [247]	2009	*Rickettsia conorii*	*R. sanguineus*	*R. sanguineus* s.s.	Southern France	Transstadial transmission
Iori et al. [248]	2010	*Babesia canis*	*R. sanguineus*	Several possible species	Italy	Transovarial transmission
Paz et al. [249]	2010	*Leishmania infantum*	*R. sanguineus*	*R. linnaei*	Minas Gerais, Brazil	Detection of the parasite DNA in larvae fed on an experimentally infected dog, as well as in unfed nymphs and adults moulting from those larvae
Razmi and Nouroozi [250]	2010	*Babesia ovis*	*R. sanguineus*	Several possible species	Mashhad, Iran	Unsuccessful transmission; no transovarial transmission
Zemtsova et al. [251]	2010	*Rickettsia conorii israelensis*	*R. sanguineus*	*R. sanguineus* s.s. or *R. linnaei*?	Oklahoma, USA ^f^	Co-feeding and transstadial transmission
Zemtsova et al. [251]	2010	*Rickettsia conorii israelensis*	*R. sanguineus*	Several possible species	Israel	Co-feeding and transstadial transmission
Otranto et al. [252]	2011	*Cercopithifilaria bainae*	*R. sanguineus*	*R. secundus* or *R. rutilus*?	Basilicata, Sicily, Italy	Third-stage larvae in naturally infected ticks
Pacheco et al. [253]	2011	*Rickettsia rickettsii*	*R. sanguineus*	*R. linnaei*	Minas Gerais, Brazil	Transmission to rabbits by bites; transstadial and transovarial transmission
Piranda et al. [254]	2011	*Rickettsia rickettsii*	*R. sanguineus*	*R. linnaei*	Rio de Janeiro, Brazil	Transmission to guinea pigs by bites; transstadial and transovarial transmission
Billeter et al. [255]	2012	*Bartonella vinsonii berkhoffii*	*R. sanguineus*	*R. sanguineus* s.s. or *R. linnaei*?	Obtained from Centers for Disease Control, Atlanta (USA origin?)	Tick infection via capillary tube feeding, but no transovarial transmission
Brianti et al. [256]	2012	*Cercopithifilaria bainae*	*R. sanguineus*	*Rhipicephalus rutilus*	Putignano, Italy	Third-stage larvae in experimentally infected nymphs and newly moulted adults (transstadial transmission)
Levin et al. [257]	2012	*Rickettsia conorii*	*R. sanguineus*	*R. sanguineus* s.s. or *R. linnaei*?	Oklahoma, USA	Transmission to dogs by bites
Socolovschi et al. [258]	2012	*Rickettsia conorii*	*R. sanguineus*	*R. sanguineus* s.s.	Algeria	Transstadial and transovarial transmission
Demoner et al. [259]	2013	*Hepatozoon canis*	*R. sanguineus*	*R. linnaei*	Minas Gerais, Brazil	No oocysts in experimentally infected ticks
Fourie et al. [260]	2013	*Ehrlichia canis*	*R. sanguineus*	*R. sanguineus* s.s.?	France	Transmission to dogs by bites; transstadial transmission
Giannelli et al. [261]	2013	*Hepatozoon canis*	*R. sanguineus*	*R. rutilus*	Putignano, Italy	Transstadial transmission
Ramos et al. [262]	2013	*Cercopithifilaria bainae*	*R. sanguineus* s.l.	*R. rutilus*	Putignano, Italy	Third-stage larvae in naturally infected ticks
Aktas et al. [263]	2014	*Babesia occultans*	*R. turanicus*	Several possible species	Turkey	Transovarial transmission
Ramos et al. [264]	2014	*Hepatozoon canis*	*R. sanguineus* s.l.	*R. rutilus*	Putignano, Italy	Mature and immature oocysts, and free sporocysts of *H. canis* in naturally infected ticks
Ramos et al. [264]	2014	*Cercopithifilaria bainae*	*R. sanguineus* s.l.	*R. rutilus*	Putignano, Italy	Third-stage larvae in naturally infected ticks
Stoffel et al. [265]	2014	*Ehrlichia chaffeensis*	*R. sanguineus*	*R. sanguineus* s.s. or *R. linnaei*?	Purchased from the Oklahoma State University (USA origin?)	Acquisition from experimentally infected dog; no subsequent transmission attempt
Moraes-Filho et al. [266]	2015	*Ehrlichia canis*	*R. sanguineus* group (tropical)	*R. linnaei*	São Paulo, Brazil	Transmission to dogs by bites and transstadial transmission
Moraes-Filho et al. [266]	2015	*Ehrlichia canis*	*R. sanguineus* group (temperate)	*R. sanguineus* s.s.	Rio Grande do Sul, Brazil; Santa Fé Province, Argentina; Montevideo, Uruguay	Unsuccessful transmission; no transstadial transmission
Bilgiç et al. [267]	2016	*Leishmania major*	*R. sanguineus*	Several possible species	Turkey	Ticks fed on experimentally infected gerbils were unable to pick up the parasite
Dabaghmanesh et al. [268]	2016	*Leishmania infantum*	*R. sanguineus*	Several possible species	Shiraz, southern Iran	Detection of the parasite DNA in eggs, larvae, nymphs and adults, which originated from females fed on a naturally infected dog
Aktas and Özübek [269]	2017	*Mycoplasma haemocanis*	*R. sanguineus*	Several possible species	Turkey	No evidence of transstadial transmission
Aktas and Özübek [269]	2017	*Candidatus* Mycoplasma haematoparvum	*R. sanguineus*	Several possible species	Turkey	No evidence of transstadial transmission
Aktas and Özübek [270]	2017	*Hepatozoon canis*	*R. sanguineus*	Several possible species	Turkey	Transstadial transmission
Giannelli et al. [271]	2017	*Hepatozoon canis*	*R. turanicus*	*R. secundus*	Valenzano, Italy	Mature oocysts in field-collected ticks
Rakhshanpour et al. [272]	2017	*Leishmania infantum*	*R. sanguineus*	Several possible species	Tehran and Alborz, Iran	Unsuccessful transmission
Aktas and Ozubek [273]	2018	*Anaplasma platys*	*R. sanguineus*	Several possible species	Turkey	Transstadial transmission
Jongejan et al. [274]	2018	*Babesia vogeli*	*R. sanguineus* (tropical)	*R. linnaei*	Taiwan	Transovarial transmission
Ipek et al. [275]	2018	*Ehrlichia canis*	*R. sanguineus*	Several possible species	Turkey	Transstadial transmission
Olivieri et al. [276]	2018	*Rickettsia massiliae*	*R. sanguineus* s.l.	Several possible species	Greece	Transmission via artificial membrane feeding
Soares et al. [277]	2018	*Babesia vitalii*^h^	*R. sanguineus* (tropical)	*R. linnaei*	São Paulo, Brazil	Unsuccessful transmission; neither transstadial nor transovarial transmission
Soares et al. [277]	2018	*Babesia vitalii*^h^	*R. sanguineus* (temperate)	*R. sanguineus* s.s.	Rio Grande do Sul, Brazil	Unsuccessful transmission; neither transstadial nor transovarial transmission
Snellgrove et al. [278]	2020	*Anaplasma platys*	*R. sanguineus* s.s.	*R. sanguineus* s.s.	Eastern Arizona, USA	Transmission to New Zealand white rabbits via blood-feeding; transstadial and transovarial transmission
Wechtaisong et al. [279]	2020	*Bartonella henselae*	*R. sanguineus*	*R. linnaei*	Taiwan	Transmission via artificial membrane feeding; transstadial transmission
Wechtaisong et al. [280]	2021	*Bartonella henselae*	*R. sanguineus*	*R. linnaei*?	Taiwan?	Transmission via artificial membrane feeding; possible transovarial transmission

^a^Studies for which we could not find the minimal amount of required information were excluded from this table. For example, Achuthan et al. [281] reported the finding of *B. canis* (probably *B. vogeli*) developmental stages in a larva, two nymphs and two adults of “*R. sanguineus*” and in two nymphs of “*R. turanicus*”. However, the full paper was not accessible to us, and we could not verify important information, e.g. developmental stages (sporozoites?), and where they were located (salivary glands?), the origin of the ticks, and if these ticks had fed on an infected dog. Similarly, we did not include unpublished observations. For example, Neitz [196] mentioned experimental observations dating back to 1952 on the transmission of *B. vogeli* (termed “*B. equi*”) by “*R. sanguineus*” from South Africa, which are not included here

^b^The pathogen species in some studies is presumed. The name of the pathogen in this table may also differ from that given in the original source owing to nomenclatural changes

^c^Inferred either from reported molecular data or from the location where ticks were collected. A question mark (?) denotes either a lack of reliable information about the origin of ticks, or ticks collected from countries (e.g. Israel) where different species of the *R. sanguineus* group coexist

^d^Data should be interpreted with caution, especially for pathogens that are not transmitted primarily by ticks (e.g. *Leishmania infantum* [282]). Transstadial and transovarial transmission in this column refer to the passage of the pathogen from one tick stage to another (from larva to nymph or from nymph to adult) or from the female tick to her offspring. In some instances, transmission to the host was confirmed after transstadial or transovarial passage. The reader should refer to the cited study for more detailed information

^e^Transovarial transmission was considered unlikely by Groves et al. [213], and the available evidence indicates that it does not occur

^g^*Rickettsia conorii* is not endemic to the USA, but Levin et al. [161] used a USA strain of *R. sanguineus* s.l. in their experimental transmission studies

^h^This piroplasm has been placed in the monospecific genus *Rangelia* in recent studies, but there is unequivocal phylogenetic evidence that it belongs to the genus *Babesia*, which is part of the *Babesia* s.s. clade [283–285]

In addition to experimental transmission studies under laboratory conditions, epidemiological evidence is fundamental to ascertain the role of ticks as vectors of a given pathogen. For example, *R. linnaei* from Brazil is a competent vector for *Rickettsia rickettsii* under laboratory conditions [254], but thus far, there is no strong epidemiological evidence supporting it as a significant vector of *R. rickettsii* in Brazil, where *Amblyomma sculptum* and *Amblyomma aureolatum* are important vectors [286–288]. On the other hand, there is convincing evidence that *R. linnaei* is the primary vector of *R. rickettsii* in Mexico [289–293]. It is difficult to ascertain whether *R. linnaei* was also involved in outbreaks of Rocky Mountain spotted fever in western Arizona, USA [294], as *R. sanguineus* s.s. is also found in this state [90, 158, 295]. Similarly, there is evidence that *R. rutilus*, a proven vector of *Cercopithifilaria bainae* [256] and *Hepatozoon canis* [261], might transmit *Babesia vogeli* and *Ehrlichia canis* in southern Italy [296, 297]. Indeed, both pathogens are prevalent in a dog shelter in the Apulia region, where *R. rutilus* was the only tick found on dogs in numerous studies conducted since 2010 [297–299].

With the exception of *R. linnaei* and *R. sanguineus* s.s., information regarding the vector role of *R. sanguineus* group tick species is scarce. Furthermore, data from the relevant studies should also be interpreted with caution. For example, some authors supposedly assessing the vector competence of “*R. turanicus*” were, in fact, dealing with *R. sanguineus* s.s. from France [243] and *R. secundus* from Italy [271]. While experimental transmission studies are limited, there are many reports of pathogen DNA detection in other tick species belonging to the *R. sanguineus* group [300]. Examples of this include *Anaplasma platys* in *R. camicasi* from Kenya [301], *R. afranicus* in Sudan [96] and *R. linnaei* in Sri Lanka [112]; Crimean-Congo haemorrhagic fever virus and *Rickettsia massiliae* in *R. guilhoni* in Senegal [302–304]; West Nile virus in *R. guilhoni* in Slovakia [305]; *Rickettsia conorii* in *R. pumilio* in territories of the former Soviet Union [306, 307]; *Rickettsia sibirica* in *R. pusillus* in Portugal [308]; and *Rickettsia hoogstraalii* in *R. rossicus* in Romania [129]. There are many other examples of DNA detection studies or even pathogen isolation (e.g. [309–312]) but listing all these studies is far beyond the scope of this review. Notably, DNA detection or pathogen isolation alone does not prove vector competence.

From a historical perspective, old publications contain relatively few mentions of unpublished results, personal communications, or observations, and may include doubtful citations. For example, Wenyon [170], in volume 2 of his celebrated book “*Protozoology, a manual for medical men, veterinarians and zoologists*”, wrote the following when referring to “*Babesia canis*” transmission: “Specimens of *R. sanguineus* brought to England by James infected English dogs” (page 1018). The origin of these ticks is unclear, but they were likely from somewhere in India. Indeed, in his publication on *Hepatozoon canis*, James [313] mentioned that one of the dogs from Guwahati (India) was infected with *B. vogeli* (referred to as “*Piroplasma canis*”). Additionally, Wenyon [170] wrote “Similarly, the writer brought to England specimens from Aleppo, which infected a dog six months later” (page 1018). It is supposed that Wenyon was referring to brown dog ticks (*R. linnaei*?) and that these ticks transmitted *B. vogeli* to a dog in England. While these facts cannot be scientifically verified, they are part of the long-standing history of brown dog ticks as vectors of pathogens in dogs.

We realize that the data in Table 3 may not be exhaustive. For example, we were unable to retrieve the full texts of some old publications (e.g. [168, 177, 195, 199, 202, 221, 289, 314]). Data from some of these works are included in Table 3 on the basis of abstracts retrieved from electronic databases or detailed information provided in key references or historical reviews on the role of ticks as vectors of pathogens (e.g. [169, 196, 315]). However, while not exhaustive, this table may represent an important resource for future studies on the vector competence of *R. sanguineus* group ticks for pathogens of medical and veterinary importance.

## Knowledge gaps

Data gathered during the past 20 years have closed some long-standing knowledge gaps concerning the *R. sanguineus* group, but also led to new questions. One of the bigger questions is that regarding the vector competence of members of this species group. For example, the available experimental and epidemiological data suggest that the importance of *R. sanguineus* s.s. and *R. linnaei* as vectors for different pathogens (e.g. *E. canis* and *H. canis*) may vary (Table 3). Similarly, it is evident that *R. linnaei* is the principal vector of *R. rickettsii* in Mexico, but that the vectorial role of this tick species elsewhere (e.g. in Arizona, where both *R. sanguineus* s.s. and *R. linnaei* coexist) needs further research. While we were able to trace the actual tick species used in some of the previous transmission studies, it is virtually impossible to determine the species used in some of the others, either because precise information on the origin of the ticks is lacking or because several species may be present in the areas from which the ticks originated. Therefore, some of the concepts proposed in previous studies may need confirmation, including the role of various species (e.g. *R. sanguineus* s.s., *R. secundus*, and *R. rutilus*) in the transmission of *R. conorii* and *R. massiliae* in the Mediterranean region.

The geographical distribution of *R. sanguineus* group ticks in America and Australia has been relatively well resolved [88–90]. However, the same cannot be said for Europe, Asia and Africa, where large-scale molecular studies are needed to understand the distributions of various species of the *R. sanguineus* group. For example, *R. afranicus*, *R. linnaei* and *R. sulcatus* and several other *Rhipicephalus* spp. not belonging to *the R. sanguineus* group are present in South Africa. Considering the various climate zones found in South Africa, it would be interesting to investigate the presence of *R. sanguineus* s.s. in the temperate zone of the country, which may have a suitable climate for this species [316–319].

Genetic data would also be valuable for tracing imported cases, such as the recent report of *R. rutilus* in a dog in Ontario, Canada [113]. This dog had a history of travel from Egypt, where *R. rutilus* is present. Myers et al. [113] also reported seven cases of dogs infested with *R. linnaei* with a history of travel or living in houses with family members who had recently travelled to countries were *R. linnaei* is present. Whether exotic species such as *R. rutilus* can establish in the Western Hemisphere remains unknown, although this should not be completely ruled out, considering the introduction and successful establishment of long-horned ticks (*Haemaphysalis longicornis* Neumann, 1901) in the USA [9].

There are still questions to be answered concerning the taxonomy of species such as *R. leporis*, *R. moucheti*, *R. pumilio*, *R. schulzei*, and *R. sulcatus.* There are no bona fide reference sequences from these species, i.e. those generated from tick samples collected from the type host at the type locality. This is an important gap that needs to be closed. For *R. schulzei*, there are virtually no publicly available DNA sequences. This species has been confounded with *R. pumilio* [46], a species whose validity has been questioned by Zahler et al. [49], who suggested that it is conspecific with *R. rossicus*. Guglielmone and Nava [320] considered *R. pumilio* provisionally valid, and we agree with this. A comprehensive integrative study of ticks from Azerbaijan, China, Iran, Kazakhstan, Kyrgyzstan, Tajikistan, Turkmenistan, and Uzbekistan could shed light on the taxonomic status of several taxa belonging to *Rhipicephalus* spp. that exist in those parts of the world. The available *cox1* sequences of *R. leporis* cannot be reliably separated from those that have now been shown to originate from *R. linnaei* (Fig. 3, Additional file 8). Available 16S rRNA sequences of *R. moucheti* from Cameroon [321] were not included in our analysis because of their short length. As Morel [321] did not state the depository of the type specimens, new tick collections from the type host (common patas monkey, *Erythrocebus patas*) at the type locality (Maroua, Cameroon) would be valuable for a better delineation of *R. moucheti*. This would allow the designation of a neotype and the production of reliable sequences (e.g. complete *cox1* or mitogenome sequences) for this species.

Similarly, a comprehensive study (e.g. including the mitogenome and controlled crossbreeding) of ticks identified morphologically as *R. hibericus* in Spain, Portugal and France would be valuable for better differentiation of this species from *R. sanguineus* s.s. This new species was described on the basis of type specimens from Spain [14], which morphologically resembled *R. turanicus* but clustered phylogenetically with *R. sanguineus* s.s*.* Millán et al. [14] also included Portugal in the distribution range of the new species. In a study carried out in Portugal, Dantas-Torres et al. [63] reported noticeable morphological variations in ticks collected from different regions and even within the same region. Among the 108 males analysed, 10 presented spiracular plates with short and large dorsal tails, which resembled those of *R. turanicus*. However, these ticks were genetically indistinguishable from those presenting spiracular plates with elongated and narrow tails on the basis of 16S rRNA gene sequences [63], i.e. three haplotypes included both ticks with typical *R. sanguineus* s.s. morphology and *R. turanicus*-like ticks. This confirmed that, at least in Portugal, these ticks are morphotypes of the same species. Millán et al. [14] reported that one of their samples clustered with *R. sanguineus* s.s. with respect to all three gene fragments, but they hypothesized that this sample was an *R. sanguineus* s.s. × *R. hibericus* hybrid.

In this context, Millán et al. [14] reported the establishment of a hybrid tick colony with adults of *R. sanguineus* s.s. from a kennel (“endophilic” strain) and adults of alleged *R. hibericus* (“exophilic” strain), which were found in a nearby area (“at a distance of no more than 150 m”). They reported that these *R. sanguineus* s.s. adults were obtained to establish the colony that provided the neotype of *R. sanguineus* s.s. as described by Nava et al. [68], so apparently both tick strains were collected in Montpelier, although this is not explicit in their text. The authors obtained an F1 (high egg production and hatchability and moulting success), but the F2 was infertile. They concluded that this colony included two species: the endophilic *R. sanguineus* s.s. and the exophilic *R. hibericus*. These data should be interpreted with caution, and new controlled crossbreeding experiments with proper morphological and phylogenetic characterization of parental ticks are recommended. The alleged exophilic *R. hibericus* could be, in fact, *R. secundus*, which is thought to be present in France [9]. We tentatively consider *R. hibericus* from Spain as a valid species, pending more comprehensive morphological and phylogenetic studies to clearly demonstrate its evolutionary separation from *R. sanguineus* s.s. in Spain and possibly other countries in the western Mediterranean region of Europe. These studies may confirm the validity of *R. hibericus* or demonstrate that it is, in fact, a morphological variant of *R. sanguineus* s.s., as has been suggested in Portugal by two independent studies [63, 322].

More than 20 years ago, it was suggested that *R. rossicus* and *R. pumilio* may be conspecifics [49, 50]. In our analysis, the 12S rRNA sequences of *R. rossicus* (accession number AF150021) and *R. pumilio* (accession number AF150023) from Beati and Keirans [50] clustered in distinct clades (Fig. 1; Additional file 6). In particular, the sequence of *R. rossicus* clustered with a sequence (accession number KJ425484) of *R. rossicus* from Romania, where this species predominates in dogs [92, 93]. Both species are presently considered valid [9], but a morphological re-examination of the males of *R. rossicus* (repository—The Natural History Museum, London), and *R. pumilio* (repository—Zoological Museum, Amsterdam [16]) would be valuable for confirming the validity of the latter.

Finally, when depositing molecular data in databases (e.g. GenBank), providing information about the collection locality and/or associated host might be of great importance. Even if the primary study did not focus on this type of information, it may still be of interest for meta-analyses or data mining studies (as performed herein). In fact, host association and geographical distribution may be particularly elucidative when a sequence is assigned to a species other than the species of the corresponding name.

## Conclusions

Ticks of the *R. sanguineus* group have long been associated with domestic animals and humans [323–325]. Archaeological data confirmed the association between *R. sanguineus* group ticks and dogs from ancient Egypt [323, 324]. Coupling these data with genetic data may shed light on the original distribution and subsequent spread of *R. sanguineus* group ticks. Unfortunately, samples may not always be available for DNA extraction, or when they are available, DNA amplification and sequencing may not always be successful. Among the ticks belonging to the *R. sanguineus* group, *R. sanguineus* s.s. and *R. linnaei* predominate in temperate and tropical regions, respectively. However, they may be found in sympatry, and other species may dominate locally (e.g. *R. rossicus*, *R. secundus*, and *R. rutilus*). The taxonomy of the *R. sanguineus* group still has some gaps to be filled by tick taxonomists, who should unite their efforts toward more cooperative taxonomic work, as was the case for the neotype designation and redescription of *R. sanguineus* s.s. [68].

From both a medical and a veterinary perspective, *R. sanguineus* s.s. and *R. linnaei* are extraordinary vectors of numerous pathogens, but other species of the *R. sanguineus* group are also competent vectors, such as *R. rutilus* in southeastern Europe. We advocate the use of DNA sequence analyses for proper molecular characterization of ticks included in any study dealing with *R. sanguineus* group ticks, even if their taxonomy is not the principal focus of the study. This would be instrumental in the better definition of the species included in each study, and should be a requirement for the study of ticks in areas where different species coexist. Similarly, the identification of *R. sanguineus* group tick species is of practical importance for studies assessing the efficacy of parasiticides [326]. The results could also be of relevance for regulatory agencies (e.g. the European Medicines Agency and the United States Food and Drug Administration), as the efficacy of products against ticks and their transmitted pathogens may vary according to species and geographical area [327]. In fact, the emergence of acaricidal resistance in *R. sanguineus* s.s. and *R. linnaei* in various regions of the world is a real problem [328–332], and further research in this field of study is warranted.

## Supplementary Information

Additional file 1: Detailed methods used for sequence retrieval and database search, data processing and compilation, sequence alignment and phylogenetic analyses, and geographical distribution.

Additional file 2: Script “GenBank2Table.py”; this Python script extracts information from GenBank files, including accession codes, organism names, nucleotide sequence sizes, countries of origin, host organisms, and associated PUBMED IDs where available. The script generates hyperlinks for accession codes and PUBMED IDs, facilitating direct access to relevant databases.

Additional file 3: Script “GenBank2Fasta.py”; this Python script extracts nucleotide sequences from GenBank files.

Additional file 4: Script “Locality2Coordinates.py”; this Python script retrieves latitude and longitude coordinates from an Excel spreadsheet containing a column with locality names (country of origin).

Additional file 5: Script in R to generate maps. This script generates maps via longitude and latitude coordinates.

Additional file 6: Detailed maximum-likelihood tree based on 635 12S ribosomal RNA (rRNA) sequences available in GenBank. The tree was inferred via alignment with 391 sites and the TIM3+F+G4 model. The coloured branches depict clades representing different members of the *Rhipicephalus* *sanguineus* group, with host associations indicated by coloured cells on the basis of GenBank data. Bootstrap values > 70 are shown. The labels in bold and colour represent reference sequences for the clade.

Additional file 7: Detailed maximum-likelihood tree based on one thousand and sixty-two 16S rRNA sequences available in GenBank. The tree was inferred via alignment with 526 sites and the TIM3 + F + G4 model. The coloured branches depict clades representing different members of the *Rhipicephalus* *sanguineus* group, with host associations indicated by coloured cells on the basis of GenBank data. Bootstrap values > 70 are shown. The labels in bold and colour represent reference sequences for the clade.

Additional file 8: Detailed maximum-likelihood tree based on 1115 *cox1* sequences available in GenBank. The tree was inferred via an alignment with 644 sites and the TIM3 + F + G4 model. The coloured branches depict clades representing different members of the *R*. *sanguineus* group, with host associations indicated by coloured cells on the basis of GenBank data. Bootstrap values > 70 are shown. The labels in bold and colour represent reference sequences for the clade.

Additional file 9: Hyperlinked spreadsheet containing information about 12S rRNA sequences of the *Rhipicephalus* *sanguineus* group used for phylogenetic and distribution analyses.

Additional file 10: Hyperlinked spreadsheet containing information about 16S rRNA sequences of the *Rhipicephalus* *sanguineus* group used for phylogenetic and distribution analyses.

Additional file 11: Hyperlinked spreadsheet containing information about *cox1* sequences of the *Rhipicephalus* *sanguineus* group used for phylogenetic and distribution analyses.

## Data Availability

All the data supporting the conclusions of this study are included in the manuscript and its additional files.
