# Supplementary material for: The Rhipicephalus sanguineus group: updated list of species, geographical distribution, and vector competence
Source: Parasit Vectors. 2024 Dec 27;17:540. doi: 10.1186/s13071-024-06572-3 (PMC11681662; doi:10.1186/s13071-024-06572-3)
Supplement: Supplementary file 1 — Additional file 1: Detailed methods used for sequence retrieval and database search, data processing and compilation, sequence alignment and phylogenetic analyses, and geographical distribution. [file 13071_2024_6572_MOESM1_ESM.pdf]

Methods

Sequence retrieval and database search

12S ribosomal RNA (12S rRNA), 16S ribosomal RNA (16S rRNA), cytochrome oxidase subunit I (*cox*1) gene sequences of *Rhipicephalus sanguineus* and related species were retrieved from GenBank using BLASTn (Basic Local Alignment Search Tool; https://blast.ncbi.nlm.nih.gov/Blast.cgi) with specific query sequences: *R. sanguineus* isolate FRA3 16S ribosomal RNA gene (MH630344.1), *R. sanguineus* isolate Samont1 cytochrome oxidase subunit I gene (MH630346.1), and *R. sanguineus* 12S ribosomal RNA gene (MH630345.1). The search parameters included an E-value = 100 and a hitlist size of 5,000. The search was limited to taxa within the *R. sanguineus* group [*Rhipicephalus sanguineus* group (taxid:578835), *Rhipicephalus afranicus* (taxid:2777527), *Rhipicephalus camicasi* (taxid:669981), *Rhipicephalus guilhoni* (taxid:765387), *Rhipicephalus linnaei* (taxid:2138177), *Rhipicephalus pusillus* (taxid:49206), *Rhipicephalus rossicus* (taxid:127008), *Rhipicephalus rutilus* (taxid:34632), *Rhipicephalus sanguineus* (taxid:34632), *Rhipicephalus sanguineus* sensu lato (taxid:2926335), *Rhipicephalus secundus* (taxid:2918497), *Rhipicephalus sulcatus* (taxid:669938), *Rhipicephalus turanicus* (taxid:34633), *Rhipicephalus hibericus* (taxid:3107788), *Rhipicephalus pumilio* (taxid:127007), *Rhipicephalus leporis* (taxid:669982), *Rhipicephalus moucheti* (taxid:2937299)]. We did not include *Rhipicephalus bergeoni* (see section "Updated list of species belonging to the *R. sanguineus* group") and *Rhipicephalus schulzei* (no sequences were available) and *R. moucheti* (sequences excluded from the analyses owing to their short size).

Data processing and compilation

Sequence data retrieved from GenBank were processed using custom Python scripts. First, the GenBank files (.gb) were converted into hyperlinked Excel spreadsheets using the script "Genbank2Table.py" (Additional file 2). In this script, each sequence record in the GenBank file was parsed using the Biopython SeqIO module [1]. Information extracted included accession codes, organism name, nucleotide sequence size, country of origin, host organism, and associated PUBMED IDs where available. The script generated hyperlinks for accession codes and PUBMED IDs, facilitating direct access to relevant databases. The final dataset was compiled into an Excel spreadsheet formatted with hyperlinks for interactive data exploration. Subsequently, nucleotide sequences were extracted from the GenBank files using "Genbank2Fasta.py" (Additional file 3). This script was employed to extract nucleotide sequences from GenBank files. Using the Biopython SeqIO, sequences were parsed from the GenBank file, and nucleotide sequences were collected based on the presence of corresponding features in the GenBank annotations. Each sequence record was formatted into FASTA format with headers containing accession codes, organism names, and country of origin where available. Finally, both the Excel spreadsheet and FASTA files were processed to ensure consistency in the number and identity of sequences.

Sequence alignment and phylogenetic analyses

To ensure uniformity in sequence length, sequences were filtered using SeqKit v.0.16.1 [2]. Sequences shorter than 300 or 350 bp were removed from the datasets of 12S rRNA/16S rRNA, and *cox*1, respectively. Sequence alignment was performed using MAFFT v. 7 [3] with default parameters. Then, sequences were trimmed using BioEdit [4] with the BLAST query sequences

as references to remove poorly aligned regions. Outgroup sequences were added to the dataset to provide context for phylogenetic analysis. Maximum likelihood phylogenetic inference was conducted using IQTREE-2 [5] with the ultrafast bootstrap (1000 replicates). The best-fit evolutive model was selected using ModelFinder [6] implemented in IQ-TREE2. Trees were edited using iTOL v.6 [7].

Geographical distribution

Geographic data for localities of interest were compiled from the Excel spreadsheet containing a column with locality names (country of origin) as available on GenBank. Geocoding was performed using a Python script ("Locality2Coordinates.py"; Additional file 4), using the geopy (https://geopy.readthedocs.io/). The geocoding procedure involved attempts to retrieve latitude and longitude for each locality. The retrieved coordinates were added to the existing spreadsheet, with new columns 'Latitude' and 'Longitude' created to store the geocoded information.

To visualize the geographical distribution of *R. sanguineus* group, we used R v.4.3.0 (https://www.R-project.org/) (see Additional file 5) for generating world maps with overlaid coordinate points. The plotting and map creation were conducted using the ggplot2 v.3.4.4 [8] and maps v. 3.4.1 (https://cran.r-project.org/package=maps) packages in R.

References

1. Cock PJA, Antao T, Chang JT, Chapman BA, Cox CJ, Dalke A, et al. Biopython: freely available Python tools for computational molecular biology and bioinformatics. Bioinformatics. 2009;25:1422-3.
2. Shen W, Le S, Li Y, Hu F. SeqKit: A cross-platform and ultrafast toolkit for FASTA/Q File

manipulation. PLoS One. 2016;11:e0163962.

3. Katoh K, Rozewicki J, Yamada KD. MAFFT online service: multiple sequence alignment, interactive sequence choice and visualization. Brief Bioinform. 2019;20:1160-6.
4. Hall TA. BIOEDIT: a user-friendly biological sequence alignment editor and analysis program for Windows 95/98/ NT. Nucl Acids Symp Ser. 1999;41:95-8.
5. Minh BQ, Schmidt HA, Chernomor O, Schrempf D, Woodhams MD, Von Haeseler A, et al. IQ-TREE 2: New models and efficient methods for phylogenetic inference in the genomic Era. Mol Biol Evol. 2020;37:1530-4.
6. Kalyaanamoorthy S, Minh BQ, Wong TKF, Von Haeseler A, Jermiin LS. ModelFinder: Fast model selection for accurate phylogenetic estimates. Nat Methods. 2017;14:587-9.
7. Letunic I, Bork P. Interactive Tree of Life (iTOL) v6: recent updates to the phylogenetic tree display and annotation tool. Nucleic Acids Res. 2024;52:W78–82.
8. Wickham H. ggplot2: elegant graphics for data analysis. Springer-Verlag New York; 2016.
