# Supplementary figures and images for: The Rhipicephalus sanguineus group: updated list of species, geographical distribution, and vector competence
Source: Parasit Vectors. 2024 Dec 27;17:540. doi: 10.1186/s13071-024-06572-3 (PMC11681662; doi:10.1186/s13071-024-06572-3)

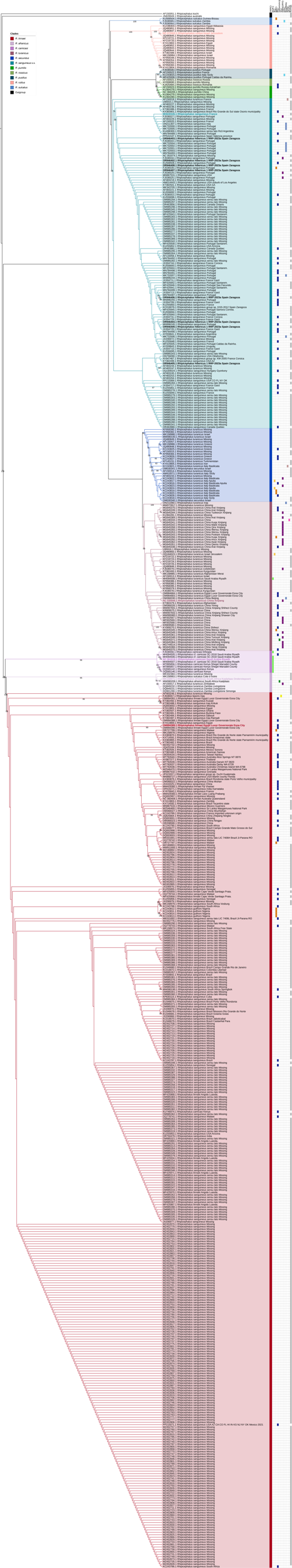

Supplement: Supplementary file 6 — Additional file 6: Detailed maximum-likelihood tree based on 635 12S ribosomal RNA (rRNA) sequences available in GenBank. The tree was inferred via alignment with 391 sites and the TIM3+F+G4 model. The coloured branches depict clades representing different members of the Rhipicephalus sanguineus group, with host associations indicated by coloured cells on the basis of GenBank data. Bootstrap values > 70 are shown. The labels in bold and colour represent reference sequences for the clade. [file 13071_2024_6572_MOESM6_ESM.pdf]
