# Supplementary material for: The Rhipicephalus sanguineus group: updated list of species, geographical distribution, and vector competence
Source: Parasit Vectors. 2024 Dec 27;17:540. doi: 10.1186/s13071-024-06572-3 (PMC11681662; doi:10.1186/s13071-024-06572-3)
Supplement: Supplementary file 7 — Additional file 7: Detailed maximum-likelihood tree based on one thousand and sixty-two 16S rRNA sequences available in GenBank. The tree was inferred via alignment with 526 sites and the TIM3 + F + G4 model. The coloured branches depict clades representing different members of the Rhipicephalus sanguineus group, with host associations indicated by coloured cells on the basis of GenBank data. Bootstrap values > 70 are shown. The labels in bold and colour represent reference sequences for the clade. [file 13071_2024_6572_MOESM7_ESM.pdf]

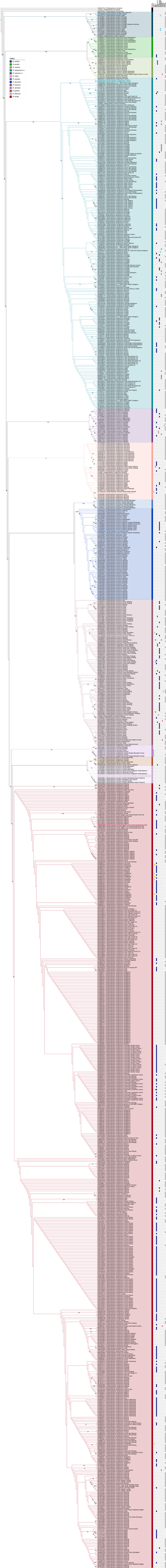
Clades
R. pusillus
R. pumilio
R. rossicus
R. sanguineus s.s.
R. turanicus s.l.
R. rutilus
R. sulcatus
R. secundus
R. turanicus
R. camicasi
R. guilhoni
R. afranicus
R. linnaei
